# Graph-CRISPR: a gene editing efficiency prediction model based on graph neural network with integrated sequence and secondary structure feature extraction

**DOI:** 10.1093/bib/bbaf410

**Published:** 2025-08-15

**Authors:** Yaojia Jiang, Bohao Li, Jiankang Xiong, Xiuqin Liu

**Affiliations:** School of Mathematics and Physics, University of Science and Technology Beijing, 30 Xueyuan Road, Haidian District, Beijing 100083, China; School of Computer Science and Engineering, Sun Yat-sen University, 132 Outer Ring East Road, Guangzhou University Town, Panyu District, Guangzhou, Guangdong 510000, China; National Center for Mathematics and Interdisciplinary Sciences, Academy of Mathematics and Systems Science, Chinese Academy of Sciences, 55 Zhongguancun East Road, Haidian District, Beijing 100190, China; School of Mathematics and Physics, University of Science and Technology Beijing, 30 Xueyuan Road, Haidian District, Beijing 100083, China

**Keywords:** CRISPR-Cas9, gene editing efficiency, secondary structure, graph neural network, on-target

## Abstract

Clustered regularly interspaced short palindromic repeats (CRISPR) gene-editing technology has transformed molecular biology. Predicting editing efficiency is crucial for optimization, and numerous computational models have been created. However, many current models struggle to generalize across diverse editing systems, often experiencing performance drops with varying conditions or systems. Additionally, most models focus on ribonucleic acid (RNA) sequence and thermodynamic features, overlooking the importance of secondary structure information. Here, we present the first graph-based model (Graph-CRISPR) that integrates both sequence and secondary structure features of single guide RNA enhancing editing efficiency prediction. Tests show Graph-CRISPR consistently surpasses baseline models across systems like CRISPR-Cas9, prime editing, and base editing. It also demonstrates strong resilience, maintaining robust performance under varying experimental conditions. This work highlights the potential of integrating sequence and structural information through graph-based modeling to enhance predictive accuracy and adaptability in gene editing applications. The datasets and source codes are publicly available at: https://github.com/MoonLBH/Graph-CRISPR

## Introduction

Clustered regularly interspaced short palindromic repeats (CRISPR) initially referred to a deoxyribonucleic acid sequence discovered as part of a bacterial defense mechanism against viral infections. Inspired by this bacterial defense mechanism, scientists have subsequently developed a series of gene-editing systems [1–3]. Over the years, scientists have focused on improving the efficiency of targeted DNA editing. To enhance editing performance, researchers have started applying various machine learning and deep learning algorithms to predict the editing efficiency of single guide ribonucleic acid (sgRNA) [4–12]. However, current approaches still face two major challenges in predicting editing efficiency.

First, existing feature engineering has not adequately represented the complex interaction networks of biological systems, particularly lacking a quantitative description of crucial conformational features such as the secondary structure of sgRNA. While these structures can enhance target specificity, excessive hairpin loops, misfolding, or overly stable conformations may reduce editing efficiency and lead to off-target effects [13, 14]. Recent advances in RNA structure prediction techniques (e.g. MXfold [15], UFold [16], SPOT-RNA [17]) now enable accurate modeling of these features.

Second, the generalizability of existing models is severely limited across different experimental platforms and cellular environments, hindering practical applications. To address these challenges, we propose an innovative approach combining graph-based representations and deep learning.

To better represent secondary structure information, we introduced graph-based data representation for the first time in the process of gene editing efficiency prediction, aiming to better integrate sequence and secondary structure information. To further leverage the advantages of graph data, we incorporated graph neural networks (GNNs) and graph attention networks (GATs) [18–20]. At the same time, we also employed the embedding vectors obtained from an RNA language model to semantically enhance the sequence features of sgRNA.

Building on this framework, we developed Graph-CRISPR, a deep learning model for CRISPR-Cas9 editing efficiency prediction. First, the model’s effectiveness was demonstrated using Kim’s datasets [4] during its development, highlighting the importance of incorporating secondary structures and embedding matrices. The model was then applied to several functional or endogenous Cas9 datasets and compared with various benchmark models. Finally, Graph-CRISPR was adapted for prime editing (PE) and base editing (BE) systems to test its cross-system compatibility. Multi-dimensional evaluations show that Graph-CRISPR performs excellently across different datasets and editing systems, effectively adapting to data generated under diverse experimental conditions.

## Materials and methods

### Dataset

In the model development and initial testing phase, the dataset we used was provided by Kim *et al.* [4] in 2019. The dataset consists of three parts, the HT_Cas9_Train training set (hereinafter referred to as Kim’s train), the HT_Cas9_Test testing set (referred to as Kim’s test), and the endogenous Endo_Cas9 set (referred to as Kim’s endo), containing 12 832, 542, and 124 sgRNA sequences respectively.

During the model generalization testing phase, we selected six datasets for comparison with other five baseline models. Three of them were derived from Wang *et al.* [8], namely WT, ESP and HF. The remaining three datasets came from public sources [21, 22], namely HCT116, HELA, and HL60.

Finally, to evaluate the adaptability (robustness) of Graph-CRISPR in the PE and BE gene editing systems, we successively used the pegRNA dataset prepared by Kim *et al.* [23] (denoted as Peg-set) and the BE system dataset from Li *et al.* [24] (denoted as Be-set). The relevant information of all the datasets used has been organized as shown in Table 1 (for more details, refer to the ‘Dataset’ section in the supplementary information).

**Table 1:** Dataset used in this study

System	Dataset name	Ref	size
Crispr-Cas9	Kim’s train	[6]	12 832
	Kim’s test	[6]	542
	Kim’s endo	[6]	124
	HF	[8]	55 603
	WT	[8]	55 573
	ESP	[8]	58 616
	HL60	[22]	2077
	HELA	[21]	8101
	HCT116	[21]	4239
Prime editing	Peg-set	[23]	48 000
Base editing	Be-set	[24]	1134

### Constructing geometry graph data

#### Mapping between nucleotide sequences and graph structures

A graph G consists of a finite non-empty set V, called vertices, and a possibly empty set E, consisting of 2-element subsets of V, called edges. Vertices are also referred to as points or nodes, while edges can also be called lines or links [25]. As mentioned in [26], the 2D characteristics of RNA secondary structure make it particularly suitable for modeling and analysis using graph theory methods. The representation of graph data has already been applied in the characterization of RNA [26, 27]. This study draws on the research ideas of sgRNA sequence graph representation and proposes an intuitive graph construction strategy, at the macro level, each 20 nt sgRNA sequence is mapped to an independent graph; at the micro level, the nucleotides in each sgRNA sequence are represented as 20 nodes in the graph, with the connectivity between nodes determined by two types of interactions: (i) ‘structural edges’ caused by non-adjacent nucleotides due to secondary structure; (ii) ‘sequential edges’ formed between adjacent nucleotides through phosphodiester bonds. This design not only retains the linear characteristics of the sequence but also effectively captures the spatial structural information of RNA.

**Figure 1:**
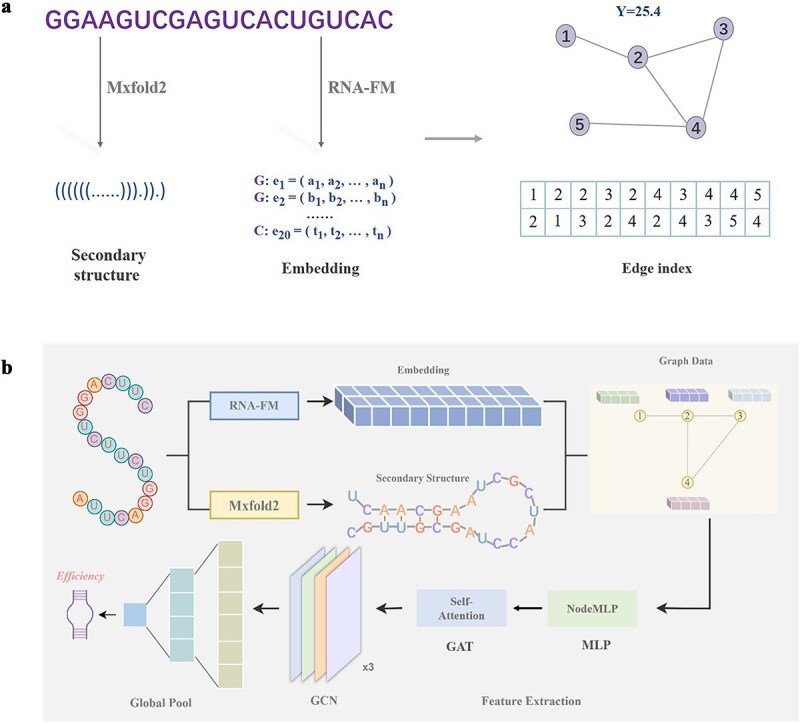
Overall process of graph data and graph model. (a) Schematic diagram of the graph data construction process. (b) Graph-CRISPR model architecture.

In Fig. 1a, we illustrate the process of converting sgRNA sequence data into graph structure data through a schematic diagram. Each sgRNA nucleotide sequence is mapped to a distinct graph structure. The arrows in Fig. 1a represent, from left to right, the process of extracting secondary structures (presented in dot-bracket notation) and embedding matrices (expressed in vector form) from 20 nucleotide sequences. Subsequently, these features are integrated to form graph data, where nucleotides serve as nodes and structural relationships serve as edges (for further details, please refer to the ‘Graph Data’ section in the supplementary information).

#### Construction of nodes

In the graph structure, each sgRNA’s 20 nucleotides correspond to 20 nodes, with each node possessing its own attributes (feature vectors). The collection of these node attributes forms the node feature matrix. Traditional models typically utilize one-hot encoding to represent sgRNA sequences; however, this method has limitations in feature representation. Here we adopted a specific RNA language pre-training model, RNA-FM [28], which is capable of delving deep into the semantic features of sgRNA sequences by vectorizing each nucleotide, thereby effectively overcoming the shortcomings of the one-hot encoding method. Consequently, replacing the traditional one-hot encoding with an embedding matrix significantly enhances the expressive capacity of the graph structure regarding information.

#### Construction of edges

In the sgRNA sequence, adjacent nucleotides interact through phosphodiester bonds, and this natural sequence connectivity is represented in the graph structure as sequential edges formed between neighboring nodes. Additionally, sgRNA may fold into secondary structures within the cell, and at this point, non-adjacent nucleotides may also interact, thereby forming structural edges. In this study, we utilized the deep learning-based RNA secondary structure prediction model Mxfold2 [29] to obtain the secondary structure information of sgRNA represented in dot-bracket notation, and constructed structural edges in the graph data accordingly.

By constructing nodes and edges, we can effectively integrate both the structural and sequence information of sgRNA, thereby forming graph data. Using graph data as input to the model, various graph-related prediction tasks can be performed.

### Architecture of Graph-CRISPR

The operational process of the GNN model in this study primarily consists of two phases. In the first phase, the original sgRNA sequence data is processed based on the RNA-FM [28] and Mxfold2 [29] preprocessing models to construct graph data input. The second phase sequentially inputs the generated graph data into the graph model composed of three modules, NodeMLP, graph attention mechanism, and graph convolution, ultimately outputting the predicted editing efficiency values. The overall architecture of Graph-CRISPR is shown in Fig. 1b (for detailed descriptions of the specific framework of each part of the model, please refer to the ‘Graph-CRISPR’ section in the supplementary information).

### Optuna hyperparameter optimization

Given the large number of hyperparameters in deep learning models, this study employs the open-source framework Optuna [30] for hyperparameter optimization. During the optimization process, not only common hyperparameters are included in the adjustment range, but also the structural choices of certain network layers are set as adjustable parameters. A total of 500 learning tasks were configured for the experiments, with mean square error (MSE) as the optimization objective, guiding the search process to identify the hyperparameter combinations that minimize MSE. For each trial, violin plots of the Spearman and Pearson correlation coefficients during the optimization process were generated to observe the stability and performance distribution of the model (Fig. S3). The specific hyperparameter search space, the hyperparameter selections, and additional information can be found in the ‘Hyperparameter Optimization and Selection’ section of the supplementary information.

## Results

### Sequence similarity filtering strengthens data independence validation

To ensure the independence between the training and test sets, this study employed MMseqs2 for the WT dataset and seqIO+Pairwise2 for the HCT116 dataset to perform sequence similarity analyses, filtering highly similar sequences with thresholds of 0.9. Analyses revealed that in the WT dataset, 98.9% of test sequences exhibited similarity values below 0.2 (low similarity range) with the training set, while only 1.1% fell into the high similarity range (0.8–1.0) (Fig. S5a). For the HCT116 dataset, 96% of sequences were within the medium similarity range (0.6–0.8), with an extremely low proportion in the high similarity range (0.8–1.0) (Fig. S5b). Quantitative statistics demonstrated that when analyzing sequence alignments between the test and training sets using the respective thresholds, the proportion of test sequences exceeding the similarity threshold corresponded to redundancy rates of 2.24% for the WT dataset, and 2.52% for the HCT116 dataset, with alignment scores predominantly concentrated in the low-value range. These results align with previously reported biological characteristics (notably the significant RNA activity heterogeneity of the WT dataset and the high cohesion of sgRNAs in the HCT116 dataset), confirming that the filtering strategy effectively mitigates the risk of data leakage (see supplementary material ‘Similarity Assessment Between Test and Training Sets and Data Filtering’ for complete analyses).

### Selection of graph data structures

In this study, the process of converting nucleotide sequences into graph data involves three key factors, (i) determining the number of vertices in the graph, (ii) selecting a secondary structure prediction model, and (iii) choosing a node embedding feature prediction model. The length of the nucleotide sequence directly determines the number of nodes in each graph. A complete synthetic sgRNA (single guide RNA) contains not only a 20-nucleotide sequence that perfectly matches the target site but also typically includes an artificially extended sequence, such as a poly-T sequence and a scaffold sequence [4]. Therefore, during the construction of graph data, we set two configurable parameters to represent the number of nodes in each graph: 20 (only the target matching sequence) and 20 + 75 (including the extended sequence, with the length of the extended sequence varying according to the data source; in the Kim’s training dataset, the length of the extended sequence is set to 75 nucleotides). Secondly, to construct the node feature matrix and structural edges of the graph data, it is necessary to generate embedding matrices and secondary structure information for each sgRNA. In terms of embedding matrix generation, this study selects two advanced RNA large language models, RNABERT [31] and RNA-FM [28], both of which have the capability to efficiently extract intrinsic features from RNA sequences. For the selection of secondary structure prediction models, UFold [16] and MXfold2 [29] are chosen as alternative models to generate structural edge connections between nodes.

**Figure 2:**
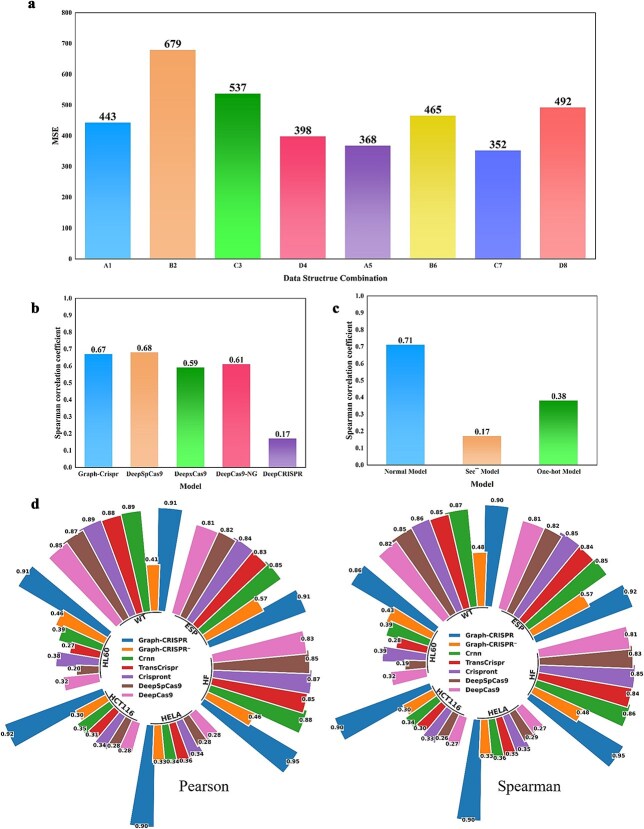
Development and evaluation of Graph-CRISPR. (a) Comparison of the MSE of eight combinations of graph data structures. (b) Performance comparison with benchmark models. (c) Ablation studies: secondary structure and embedding features. (d) Comparison with five benchmark models and ‘graph-CRISPR^**−’**^—model without pre-training.

Based on the three aforementioned aspects, we conducted experiments on the graph data structure. The experimental configuration is detailed in Table 2 (for more details, refer to the supplementary materials section ‘Selection of Graph Data Structures’). By systematically comparing the performance of different data combinations on the test set (Fig. 2a), we identified that combination C7 exhibited the optimal predictive performance, achieving the lowest MSE (MSE = 352.34) on the valid set. To further investigate the model training dynamics, we plotted the variation curves of Spearman correlation coefficients for combination C7 on both the training and valid sets as the training epochs increased (Fig. S8). The results demonstrated that after 20 training epochs, the model performance metrics gradually converged and stabilized. Notably, this combination achieved an average Spearman correlation coefficient of 0.62 on the valid set, indicating reliable predictive capability.

**Table 2:** Combinations of preprocessing models

	75 + 20 bp	20 bp
UFold+RNA-FM (A)	mse1	mse5
UFold+RNABERT (B)	mse2	mse6
MXfold2 + RNA-FM (C)	mse3	**mse7**☑
MXfold2 + RNABERT (D)	mse4	mse8

A–D represent four alternative combinations of secondary structure + embedded feature prediction model;75 + 20 bp and 20 bp represent the number of graph data nodes that can be selected.

### Graph-CRISPR model training and testing

After selecting the graph data structure, we employed the Optuna hyperparameter optimization method to identify the optimal hyperparameter combination for the model. Based on this combination, we developed the graph model. We conducted two-fold cross-validation on Kim’s train set and achieved an average Spearman and Pearson correlation coefficient of 0.94 and 0.95 on the validation set respectively (Fig. S9). This training result outperformed the performance reported in the same step of Kim *et al.* [6] (r = 0.77). Subsequently, we performed a small-scale model validation test on Kim’s endo dataset, which was used for model fine-tuning and evaluation in Kim *et al.* [4]. We selected DeepCRISPR [32] and three models (DeepSpCas9, DeepXcas9, and DeepCas9-NG) extracted from the DeepSpCas9 package [4] for comparison. The results indicated that the graph model developed in this study performed comparably to the DeepSpCas9 model on the test set, with a Spearman correlation coefficient of 0.67 for our graph model and 0.68 for Kim’s model. Both models significantly outperformed DeepCRISPR (0.17) (Fig. 2b).

This preliminary validation demonstrates that, unlike the traditional approach of processing nucleotide sequences using one-hot encoding, the use of a graph-based data structure—consisting of nodes and edges—and the integration of a GNN framework to predict gene editing efficiency is both feasible and effective. Given that this model is constructed based on graph data and a GNN, we have named it Graph-CRISPR.

### Feature importance evaluation

To explore the key roles of sgRNA secondary structure and RNA language model embedding in Graph-CRISPR, we conducted a feature importance evaluation experiment using the control variable method on the Kim’s test set. In the experiment, we retrained two models: one model removed the secondary structure edges and retained only the sequence edges; the other model replaced the node embedding feature matrix with one-hot encoding. Both models were tested alongside Graph-CRISPR on the Kim’s test set, with the test results shown in Fig. 2c.

The experimental results indicate that the standard Graph-CRISPR model yielded a Spearman correlation coefficient of 0.71 on Kim’s test set. In contrast, when the secondary structure edges were removed while retaining only the sequential edges (as detailed in ‘*Construction of Edges*’ section), the model’s performance exhibited a significant decline, with the Spearman correlation coefficient dropping to 0.17. Similarly, when the entire edge structure was retained but the node embedding matrix was replaced with a one-hot encoding matrix, the Spearman correlation coefficient also showed a marked decrease, falling to 0.38 on the test set.

### Assessing model generalizability on independent datasets

To further assess the robustness and predictive accuracy of the model across different datasets, we evaluated it on six independent test sets: WT, ESP, HF, HCT116, HELA, and HL60 (for detailed descriptions, refer to the ‘Dataset’ section). Additionally, we compared it with five baseline models: DeepSpCas9 [4], Transcrispr [7], DeepCas9 [10], CRISPRont [11], and CrnnCrispr [12].

Initially, we directly employed the optimal model parameters obtained through Optuna optimization on these six independent test sets and recorded the corresponding test results, which are presented in Table 3 and Table 4 under the label ‘Graph-CRISPR^**−’**^ (where ‘**−**’ indicates the non-pretrained version of the model). At the same time, we also observed that due to differences between datasets, the baseline models were fine-tuned before evaluation on specific test sets. To ensure methodological rigor, we also applied this pre-training strategy to the graph-based model, and the pretrained model is referred to as Graph-CRISPR.

**Table 3:** Comparison of Spearman correlation coefficients on six test sets.

	HL60	HCT116	HELA	HF	ESP	WT
Graph-CRISPR	**0.859**	**0.904**	**0.889**	**0.945**	**0.920**	**0.893**
Graph-CRISPR^−^	0.432	0.300	0.325	0.477	0.570	0.477
Crnn	0.389	0.335	0.354	0.859	0.852	0.867
TransCrispr	0.282	0.297	0.349	0.839	0.841	0.849
CRISPRont	0.394	0.333	0.348	0.851	0.846	0.862
DeepSpCas9	0.191	0.258	0.287	0.833	0.822	0.846
DeepCas9	0.323	0.268	0.271	0.814	0.813	0.815

Bold values indicate the highest correlation coefficient achieved for each test set.

**Table 4:** Comparison of Pearson correlation coefficients across six test sets.

	HL60	HCT116	HELA	HF	ESP	WT
Graph-CRISPR	**0.907**	**0.923**	**0.904**	**0.947**	**0.913**	**0.914**
Graph-CRISPR^−^	0.458	0.300	0.328	0.455	0.568	0.405
Crnn	0.386	0.346	0.344	0.875	0.846	0.891
TransCrispr	0.273	0.312	0.355	0.853	0.834	0.876
CRISPRont	0.383	0.343	0.339	0.866	0.836	0.886
DeepSpCas9	0.197	0.277	0.281	0.848	0.815	0.869
DeepCas9	0.315	0.276	0.276	0.833	0.806	0.853

Bold values indicate the highest correlation coefficient achieved for each test set.

As shown in Fig. 2d, Tables 3, and Table 4. these charts visually present the pre-training results of our model compared to direct prediction results, as well as the performance metrics of the benchmark models (these metrics were obtained after pre-training). The tables indicate that under the Graph-CRISPR^**−**^ configuration (non-pretrained model), graph-based models have already surpassed some pretrained benchmark models on the HCT116 (R = 0.3, r = 0.3), HELA (R = 0.325, r = 0.328), and HL60 (R = 0.432, r = 0.458) datasets. When switching to the fully pretrained Graph-CRISPR model, it achieved higher Spearman and Pearson correlation scores across all six datasets, significantly outperforming five benchmark models. For in-depth discussions on the pre-training strategy and its generalization capabilities, please refer to the ‘Assessing Model Generalizability on Independent Datasets’ section in supplementary materials.

### Statistical significance analysis and robustness test

To systematically evaluate the performance and robustness of the Graph-CRISPR model, we selected HCT116 (representing small-scale datasets) and WT (representing large-scale datasets) as testing platforms to balance computational efficiency and data diversity requirements. Three progressive analyses were conducted. Firstly, a permutation test was employed to assess whether the model’s predictive performance significantly exceeds random prediction levels (specific methodologies are detailed in the ‘Model Prediction Permutation Test Validation’ section). The permutation test results demonstrated that the predictive performance of the Graph-CRISPR model on both the WT and HCT116 datasets significantly exceeded random prediction (*P* < .05). This confirmed that the model’s predictive performance achieves high statistical significance (Fig. S4). Secondly, independent repeated training strategies were implemented combined with visual analysis of training loss curves on these two datasets (Fig. S10) to systematically verify the model’s stable convergence characteristics under varying initialization conditions; Finally, a high-standard test set was constructed based on sequence similarity screening, and the model’s predictive performance was evaluated on this rigorously filtered dataset (Fig. S6) (complete arguments are detailed in the supplementary material section ‘Predictive Performance on Filtered Test Sets’). The collective results of these three analyses conclusively demonstrate that the model’s outstanding performance cannot be attributed to data similarity biases, thereby providing robust support for the reliability of Graph-CRISPR (complete arguments are detailed in the supplementary material section ‘Similarity Assessment Between Test and Training Sets and Data Filtering’).

### Graph-CRISPR compatibility testing across multiple editing systems

This section further explores the compatibility and adaptability of graph-based models in response to changes in gene editing systems. Specifically, we evaluate the performance of Graph-CRISPR in two additional gene editing systems: prime editing and base editing.

### Performance evaluation of Graph-CRISPR on prime editing system

First, we selected the PE system, which resembles the Crispr-Cas9 system. This system enables precise insertion, deletion, or replacement of DNA by coupling a reverse transcriptase with a Cas9 nuclease variant, without relying on the cell’s repair mechanisms.

**Figure 3:**
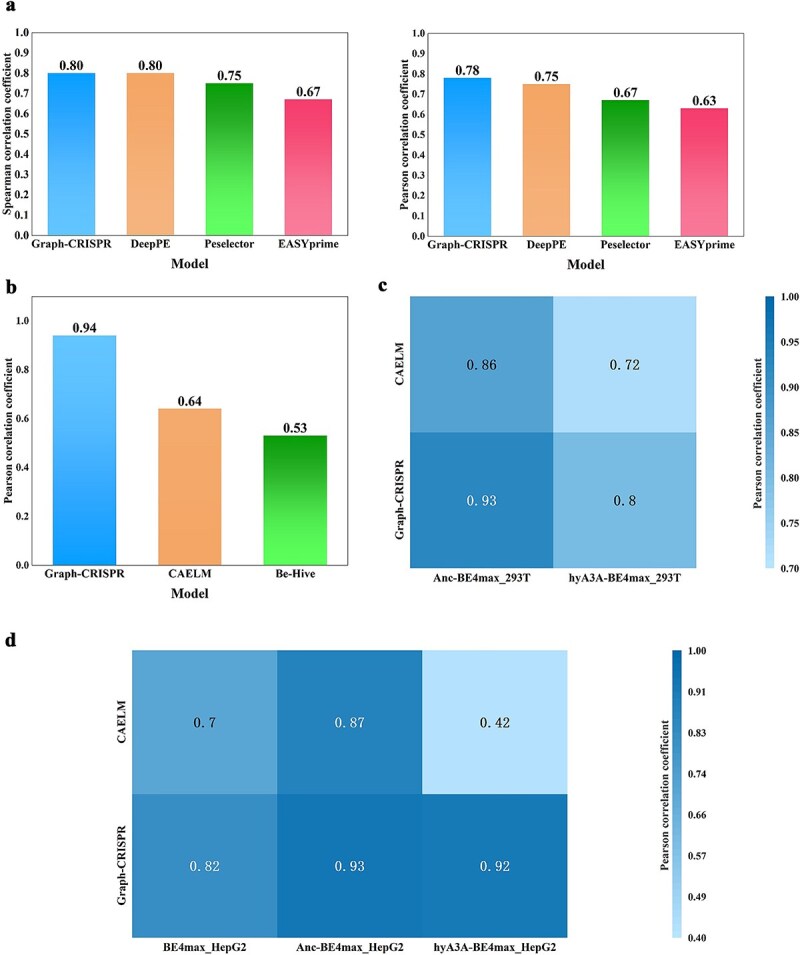
Cross-system compatibility and robustness testing of Graph-CRISPR. (a) Comparison of Spearman and Pearson correlation coefficients on PE test data across four models. (b) Pearson performance of the three models. (c) Prediction performance of the graph model and CAELM on Anc-BE4max and hyA3A-BE4max in Hek293T cells. (d) Prediction performance of the graph model and CAELM on BE4max, Anc-BE4max, and hyA3A-BE4max in HepG2 cells.

To assess the model’s performance, we selected the Peg-set dataset (as detailed in the ‘Dataset section’) as the benchmark and employed standard dataset partitioning and cross-validation strategies to avoid overfitting risks, splitting the dataset into 85% for cross-validation training and 15% for testing. We compared Graph-CRISPR with three mainstream pegRNA efficiency prediction models: DeepPE [23], easyPrime [33], and PEselector [34]. noting that the development dataset for DeepPE is the same as our benchmark dataset. The results indicate (Table 5, Fig. 3a) that Graph-CRISPR achieved a Spearman correlation coefficient of 0.8, which matches the best-performing baseline model DeepPE; in terms of Pearson correlation coefficient, Graph-CRISPR outperformed all baseline models with a score of 0.78.

**Table 5:** Comparison of model metrics on the PE system.

	Graph-CRISPR	DeepPE	Peselector	EASYprime
Spearman	**0.8**	**0.8**	0.75	0.67
Pearson	**0.78**	0.75	0.67	0.63

Bold values indicate the highest metric for Peg-set dataset.

### Performance evaluation of Graph-CRISPR on base editing system

Unlike CRISPR-Cas9 and PE systems, base editing achieves precise genetic modifications by directly replacing specific bases. This study selects the Be-set dataset as the foundational dataset (details can be found in the ‘Dataset’ section). Based on this dataset, relevant research developed the deep learning model CAELM [24], using the Pearson correlation coefficient as an evaluation metric. We strictly followed the training protocol of CAELM, utilizing the Be-set dataset for transfer learning with Graph-CRISPR. Additionally, the Be-Hive [35] (https://www.crisprbehive.design/) model from the original research was also included for performance comparison. Figure 3b illustrates the Pearson correlation coefficients of the three models. The results demonstrate that Graph-CRISPR achieved a Pearson correlation coefficient of 0.94 on the Be-set dataset, significantly outperforming CAELM (0.64) and Be-Hive (0.53).

**Figure 4:**
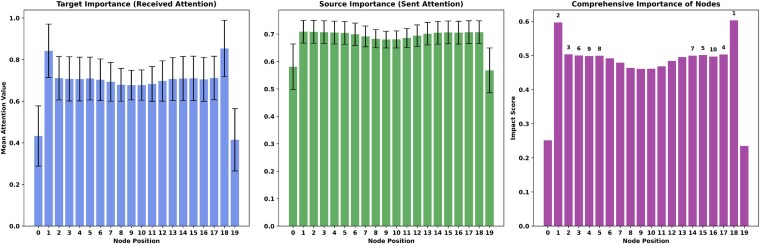
Importance ranking of positions. (a) Importance of target nodes (the total attention scores received by each node). (b) Importance of source nodes (the total attention score emitted by each node). (c) The importance of comprehensive nodes (hub nodes with high influence).

In the field of base editing, there are also various subtypes of editing systems. In the CAELM study, in addition to the basic BE4max system, two BE4max-derived editing systems were involved: Anc-BE4max and hyA3A-BE4max. The original study selected these two derivative systems along with the BE4max system, and conducted gene editing experiments using a small subset extracted from the Be-set dataset across two different cell lines. This resulted in the generation of five groups of data sets with distinct markings, which were used to test CAELM. We conducted a similar evaluation of Graph-CRISPR on these heterogeneous datasets following the training settings described in the original study. The five heterogeneous datasets and their relevant experimental details are outlined in detail in the ‘Robustness Experiments’ section of the supplementary information.

As shown in Fig. 3c and d, heatmap blocks clearly demonstrate the consistent and robust performance of Graph-CRISPR in commonly used Hek293T (r = 0.93 and 0.8) and HepG2 cells (r = 0.82, 0.93 and 0.92). Through multiple evaluation experiments, it can be concluded that the Graph-CRISPR model exhibits strong cross-system compatibility, adapts effectively to diverse gene editing systems, and demonstrates high adaptability across various gene editing datasets. Furthermore, in experiments involving different cell lines, Graph-CRISPR mitigates external interference, maintaining predictive stability and accuracy while adapting to experimental condition-induced disturbances.

### Graph-CRISPR interpretability validation through multi-dimensional analysis

To validate the decision reliability of the GNN model, we employed a multi-dimensional interpretability analysis strategy. Based on the Kim’s train set, we first conducted a preliminary analysis of the model’s dependence on graph structural features through the attention heatmap of the GAT module (Fig. S7 available online at http://bib.oxfordjournals.org/) and node functional classification assessment (Fig. 4). The results revealed that the predicted key sites were primarily concentrated in the functional regions of sgRNA—specifically the 5′ seed region (positions 2–6) and the 3′ near-PAM region (positions 15–19)—which showed high consistency with established biological evidence [22, 36–39].

Furthermore, we introduced GNNExplainer [40] to perform fine-grained analysis of the model’s decision-making process. Notably, at critical RNA sequence positions (e.g. positions 18 and 2 in 0–19), both methods consistently identified highly important nodes. This multi-modal interpretability analysis not only verifies the robustness of Graph-CRISPR’s predictive results but also provides novel biological insights into the model’s decision-making mechanism from the perspective of local subgraph structures (see ‘GNNExplainer based interpretation’ in the Supplementary materials for details).

## Conclusion and discussion

To address the limitation that previous models failed to fully consider secondary structure features, this study innovatively employs a graph-based data structure to represent sgRNAs—wherein each node corresponds to a nucleotide, and edges denote the connections between nodes, reflecting the formation of secondary structures. Based on this framework, we developed a GNN model named Graph-CRISPR for gene editing efficiency prediction.

Experimental validation demonstrated that incorporating secondary structure information significantly improves model performance, when preserving edge connections while replacing node features with one-hot encoding, the Spearman correlation coefficient showed a significant decline (Fig. 2c), confirming the necessity of combining secondary structure data with embedding matrices.

Moreover, the model demonstrates superior performance compared to baseline models across multiple generalization test sets (Fig. 2d), three editing systems, and heterogeneous datasets (Fig. 3). Notably, a significant performance gap was observed between base editing (BE; r = 0.94) and prime editing (PE, r = 0.8) systems (Fig. 2a and b). The performance gap may stem from the additional functional complexity of pegRNA (e.g. serving as a reverse transcription template), which current secondary structure models cannot fully capture. Notably, although the model was not optimized for the PE system, it still achieved a correlation of 0.8, demonstrating its capability to capture common features across different editing systems.

Future research could focus on the following aspects. First, in sgRNA graph construction, multiple nucleotides may be consolidated into single nodes based on local structural features (rather than individual nucleotides) to simplify the architecture (e.g. the RAG method [27]), though further validation is needed for its applicability to short RNAs (20 bp). Second, regarding edge feature design, a key question is whether sgRNA secondary structure edges possess quantifiable physicochemical properties (e.g. base stacking energy, phosphodiester bond torsion angles). Although edge feature representations in protein graph models [41, 42] provide valuable references, sgRNA structural edges may lack distinct biophysical characteristics, which will be a critical direction for future optimization.

In summary, this study establishes Graph-CRISPR as a versatile and high-performance deep learning framework for predicting sgRNA editing efficiency, demonstrating robust compatibility across gene editing systems and exceptional accuracy in target prediction. These findings highlight the transformative potential of graph-based architectures for gene editing-related predictions. Integrating adaptive GNNs with graph-based representations could further expand computational tool capabilities in this field. While our framework successfully generates biologically meaningful hypotheses, its reliance on existing datasets and algorithmic modeling necessitates wet-lab validation to confirm predictions—a critical direction for future research.

**Key Points**

- Incorporating secondary structure features with graph data and graph models: This study breaks through the traditional sequence data structure and innovatively constructs a graph-based representation system for single guide ribonucleic acid (sgRNA), enabling more precise feature encoding of sgRNA. On this basis, the Graph-CRISPR model was developed, which significantly improves the accuracy and robustness of sgRNA editing efficiency prediction.
- Enhancing sgRNA representation with pre-trained RNA embeddings: The embedding information obtained from an RNA language model is used to replace one-hot encoding as the node features, allowing for a more refined representation of sgRNA features and providing richer semantic information for subsequent deep learning and model parameter updates.
- Cross-system compatibility: Graph-CRISPR demonst-rates good cross-system compatibility among the three gene editing systems. Moreover, when confronted with heterogeneous datasets caused by external disturbances, the model maintains robust stability, further validating its resilience and generalizability in diverse data environments.

## Supplementary Material

supply_bbaf410

Fig_S1_bbaf410

Fig_S2_bbaf410

Fig_S3_bbaf410

Fig_S4_bbaf410

Fig_S5_bbaf410

Fig_S6_bbaf410

Fig_S7_bbaf410

Fig_S8_bbaf410

Fig_S9_bbaf410

Fig_S10_bbaf410

Table_S1_Hyperparameter_Optimization_Parameter_Combination_Space_bbaf410

Table_S2_Final_Selection_of_Hyperparameter_bbaf410

Table_S3_Test_Dataset_Split_bbaf410

Table_S4_Statistical_analysis_for_the_WT_and_HCT116_bbaf410

Table_S5_Analysis_for_the_Importance_of_Position_bbaf410

## Data Availability

All datasets used in this study can be found and downloaded from the original references and their supplementary materials. Specifically, all relevant datasets from Kim [6, 23] are available in the original articles and their supplementary materials of ‘DeepSpCas9’ and ‘DeepPE’. About the six independent test sets, WT, ESP, and HF can be found in reference [8], while HCT116, HELA, and HL60 are available in [21, 22], and Be-set can be downloaded from reference [24]. All data- sets used in this study have been uploaded to https://github.com/MoonLBH/Graph-CRISPR.
