# Supplementary material for: Graph-CRISPR: a gene editing efficiency prediction model based on graph neural network with integrated sequence and secondary structure feature extraction
Source: Brief Bioinform. 2025 Aug 15;26(4):bbaf410. doi: 10.1093/bib/bbaf410 (PMC12354951; doi:10.1093/bib/bbaf410)
Supplement: supply_bbaf410 [file supply_bbaf410.docx]

Graph-CRISPR: A gene editing efficiency prediction model based on Graph Neural Network with integrated sequence and secondary structure feature extraction

Yaojia Jiang^1^, Bohao Li^2^, Jiankang Xiong^3^ and Xiuqin Liu^1,*^

^1^ School of Mathematics and Physics, University of Science and Technology Beijing, Beijing 100083, China

^2^ School of Computer Science and Engineering, Sun Yat-sen University, Guangzhou 510000, China

^3^ National Center for Mathematics and Interdisciplinary Sciences, Academy of Mathematics and Systems Science, Chinese Academy of Sciences, Beijing 100190, China

* Corresponding author: [b0001428@ustb.edu.cn](mailto:b0001428@ustb.edu.cn)

**Supplementary Information**

**Dataset**

Kim's relevant datasets were used for training and preliminary testing of our model. Kim's endo, which is independent of the training set and composed entirely of endogenous human gene loci, includes both coding and non-coding regions, totaling 124 sequences. Kim's test includes genes at 124 endogenous loci (Kim's endo) as well as randomly generated synthetic sequences, totaling 542 nucleotide sequences. We use Kim's train to train the model and validate it on the Kim's endo and the Kim's test.

The WT, HF, and ESP datasets were obtained from Wang et al. [8]. In this study, they constructed a plasmid library targeting over 20,000 genes, which was tested in three human cell environments capable of expressing Cas9 (two of which are high-specificity variants of SpCas9: eSpCas9 and SpCas9-HF1, abbreviated as WT, ESP, and HF). For each gene, the top 3 to 4 sgRNAs were selected, resulting in a dataset of more than 80,000 oligonucleotide plasmids. Following delivery experiments and deep sequencing, reads with counts greater than 100 were selected, yielding over 50,000 reads from each of the three cell environments. The remaining three dataset came from public sources [21, 22], namely HCT116, HELA, and HL60. These three datasets, together with the aforementioned dataset from Wang et al. [8], will form the test set used in the generalization testing phase of the model.

The Peg-set is derived from the Prime Editing (PE) system. This dataset includes 24 combinations of PBS and RTT lengths (six PBS lengths: 7, 9, 11, 13, 15, 17 nucleotides × four RTT lengths: 10, 12, 15, 20 nucleotides), corresponding to 2,000 guide-target sequence pairs, totaling 48,000 pegRNA-target sequence pairs (= 24 × 2,000). And the Be-set is an experimental dataset generated based on the Base Editing (BE) system. which focused on CBE single-base editing. This dataset employed the BE4max editing system and involved 209 target loci, resulting in 1,134 entries with effective editing outcomes.

**Graph Data**

Graph data originates from Euler's work and graph theory. Over the past decade, the use of graph data to represent relationships between individuals or communities has gained increasing attention, making it a popular type of database. A graph consists of nodes, edges (representing relationships between nodes), and labels. Each node has attributes (node features), and edges, which can also have features and directions, represent the relationships between nodes. Edges can be directed or undirected, and the label typically corresponds to the entire graph, reflecting properties of the original data.

Traditionally, gene editing prediction relies on nucleotide sequence data. In this study, we introduce the use of graph data structures to predict editing efficiency. Our source data remains gRNA nucleotide sequences. Considering that gRNA can form secondary structures and nucleotide sequences can be naturally represented as nodes, we convert the sequence data into graph structures. Specifically, each gRNA sequence corresponds to one graph.

For example, a 20-nucleotide gRNA sequence is transformed into a graph, where each nucleotide corresponds to a node. If there is an interaction between any two nucleotides, an edge is considered to exist between them. In the graph data, edges are represented as sequential edges and secondary structure edges between nodes (both undirected). The label of each graph corresponds to the indel rate associated with the gRNA sequence. Our goal is to perform regression prediction of the indel rate, making this being a graph-level regression task. Fig.S1 illustrates the overall research approach.

**
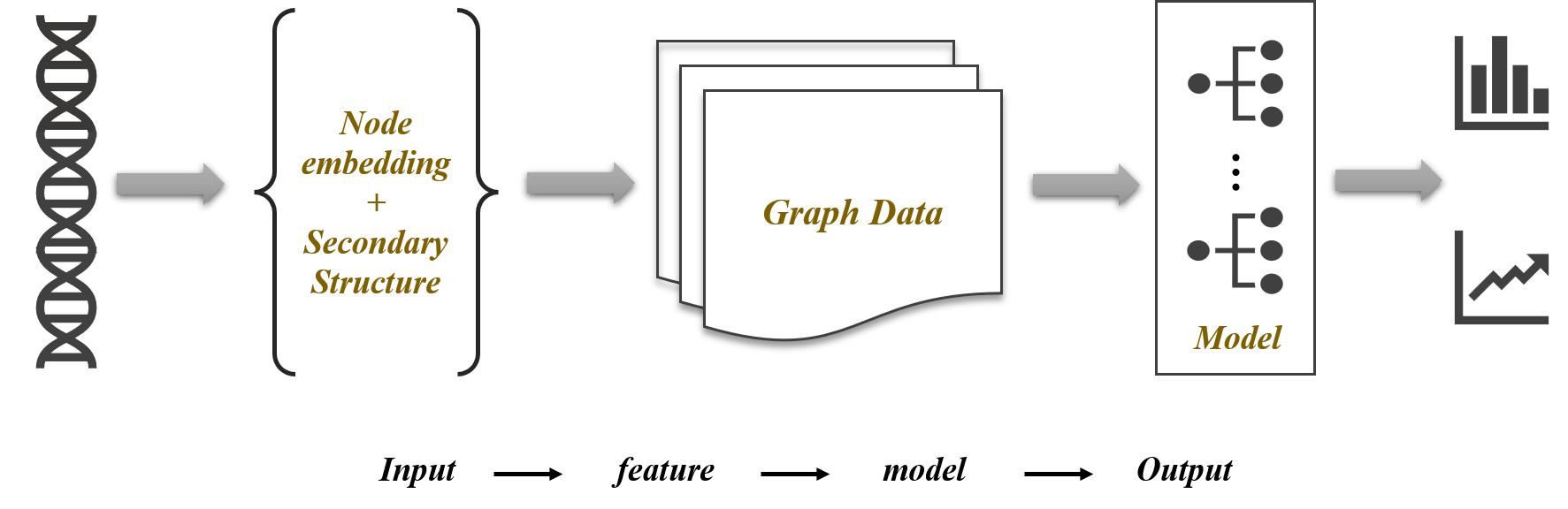
**

**Fig.S1 Workflow from input to output.** Data processing and prediction workflow.

**Graph-CRISPR**

Here, we will provide a detailed explanation of each module in Graph-CRISPR.

*MLP and Attention layers*

The NodeMLP layer rearranges the pre-trained embedding feature matrix without altering the input dimension and further integrates the node features. The output of this module is then passed as input to the multi-head attention layer. The multi-head attention layer generates multiple updated state matrices using distinct shared weights, where each attention head is composed of a Graph Attention Network (GAT). This network utilizes a self-attention mechanism to dynamically assign weights to the neighboring nodes of the central node, facilitating more flexible and precise information aggregation, thus updating the state of each node. The multiple state matrices generated by the multi-head attention layer are subjected to a concatenation layer for processing, which integrates the information from different attention heads and generates the final output matrix. This matrix is then passed as input to the convolutional module. (more detail about GAT layer can be found in next section" Graph Attention Mechanism Layer Structure").

*Graph Convolutional Neural Networks*

The third module is the Graph Convolutional Network (GCN) layer, which functions as the output layer of the entire model. It receives the state feature matrix produced by the GAT layer. The GCN layer further aggregates these feature matrices to generate predictions for editing efficiency. This aggregation is performed through three sets of GCN convolution operations (GCNConv). Subsequently, the processed feature matrix undergoes further processing via a global pooling layer and a linear layer, ultimately mapped to a single numerical value (predicted value), resulting in the prediction of editing efficiency.

Different types of graph convolutional layers primarily differ in their methods of information propagation and aggregation. Therefore, in the Optuna tuning module (It will be explained in section "Optuna hyperparameter optimization" in the supplementary), both convolution layers and pooling layers are treated as tunable hyperparameters. We have designed four different convolution operations for the graph convolutional layer to facilitate the optimization process, while the pooling layer offers two options. (For details, please refer to section "Hyperparameter Optimization and Selection" in the supplementary.) Since this study involves a graph-level prediction regression task, it is essential to aggregate the features of all nodes into a single vector, which represents the global information of the entire graph. To achieve this, we add a global pooling layer before the final fully connected layer, designed to aggregate the features of all nodes into a single vector. During the global pooling process, we provide two pooling options: average pooling and sum pooling. Through hyperparameter optimization using Optuna, we identify the optimal combination in the configuration mentioned above to optimize the model's performance.

The differences between various types of convolutional layers lie in how they aggregate the neighboring node features during this process. In the convolutional operation we selected, the update formula for the features of the central node is as follows:

Let G = (V, E) represent a graph, where V denotes the set of all nodes in the graph and E denotes the set of all edges. The feature vector of node *v* at the *l*-th layer is represented as $h_{v}^{(l)}$. Thus, the update formula can be expressed as:

$h_{v}^{(l+1)}=\sigma(\sum_{u\in N(v)\bigcup\{v\}} \frac{1}{\sqrt{d_{v}d_{u}}}W^{(l)}h_{u}^{(l)})$.

Here, $d_{v}$and $d_{u}$ represent the degrees of nodes *v* and *u*, respectively. *N(v)* denotes the set of neighboring nodes of node *v*. $W^{(l)}$ is the weight matrix at the *l*-th layer, which acts on each node's features and indicates shared weights, as all nodes within the same layer use the same weight matrix. $\sigma(\cdot)$ represents the non-linear activation function, which is included as a tunable parameter in Optuna for hyperparameter optimization in this study. Within the activation function, the inner part of the formula aggregates information from both the neighboring nodes and the current layer of the node itself. The factor $\frac{1}{\sqrt{d_{v}d_{u}}}$ normalizes the features of the neighboring nodes, ensuring that differences in degree do not lead to imbalance in the aggregation process. The outer non-linear activation function enhances the model's expressiveness.

Our convolutional module is constructed in a fixed pattern, where the node feature matrix sequentially enters four components: a graph convolutional layer, activation layer, dropout layer, and pooling layer (Fig.1B). This module is repeated three times, and its output is passed to the readout module. The readout module comprises a convolutional layer, a dropout layer, a global pooling layer, and a fully connected layer, which ultimately maps the output to a single numerical value representing the predicted indel frequency.

**Graph Attention Mechanism Layer Structure**

The illustration of central node information aggregation is shown below, where$h_{1}$represents the current state of the central node, and $h_{1}^{'}$ represents the updated state vector of the central node. $h_{2}$and $h_{3}$ represent neighboring nodes of the central node. As shown in the Fig.S2, updating the

central node's subsequent state requires aggregating information from its neighboring nodes and, optionally, its own previous state.

**
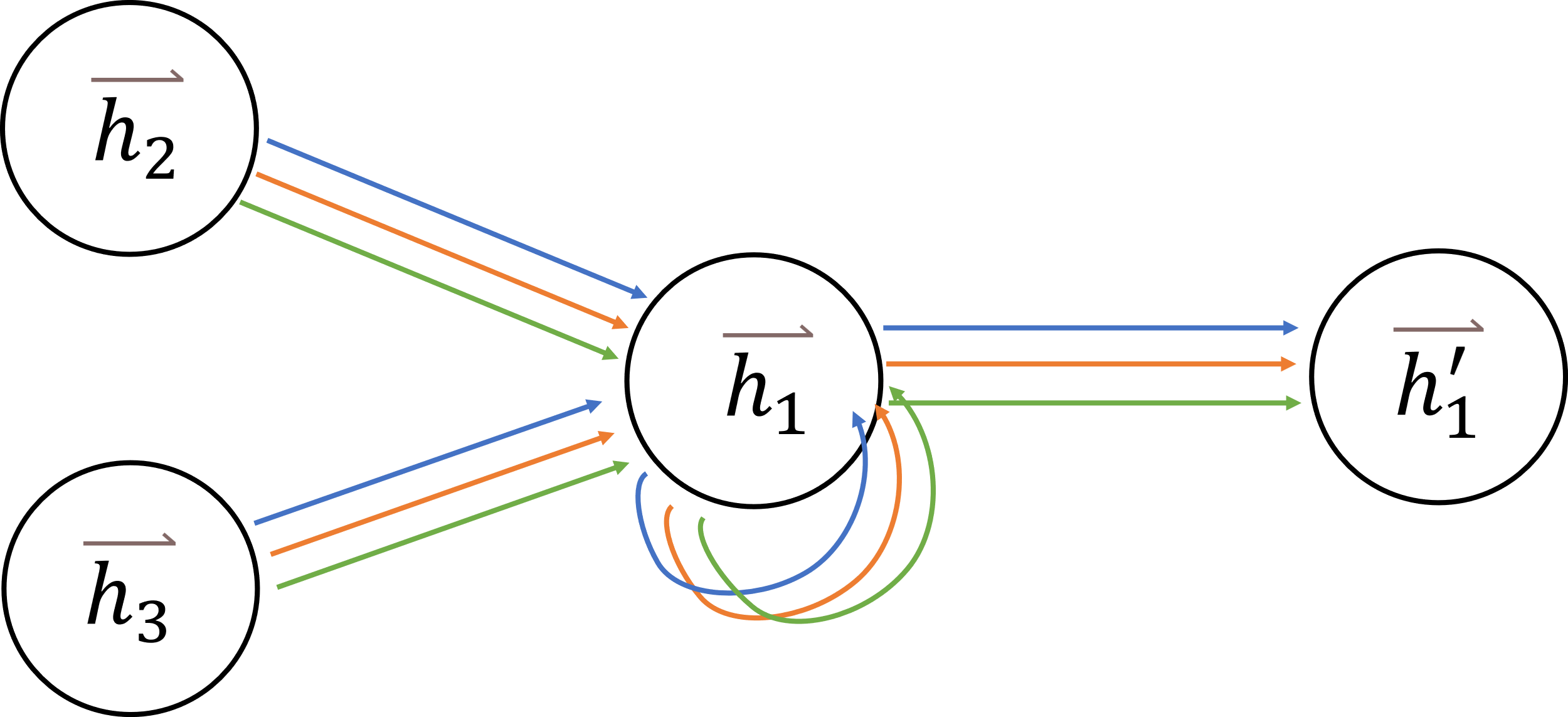
**

**Fig.S2 Illustration of central node update.** State update process of a central node ($h_{1}$ to $h_{1}^{'}$) through updating of neighboring nodes ($h_{2}$ , $h_{3}$).

The multi-head attention unit consists of two main types of attention layers: First, a multi-head feature extraction layer is created. In this layer, each attention head takes as input, the output dimension of the NodeMLP layer, denoted as [batch_size, 20, in_features]. Each head outputs features with dimensions [batch_size, 20, hidden_features], where the hidden layer dimension is manually set. For simplicity, batch_size is omitted in subsequent dimension notations. The state matrices$h$are derived by setting the number of heads to capture the outputs of different attention heads. Then, a readout attention layer is used as the output layer of this unit. This layer concatenates the state matrices obtained by each attention head, with the input feature dimensions being [20, hidden_features×gat_heads], and restores the output feature dimensions back to hidden_features.

The formula for calculating the attention scores is as follows.

We use $h_{i}$ and $h_{j}$, where $h_{i}$∈$R^{f}$ to represent each node's feature vector. A shared weight matrix W ∈$R^{fXf}$ is used to perform a linear transformation on the feature vectors. Then, a self-attention mechanism is introduced to calculate the attention coefficient α${：R}^{f}\times R^{f}\to R,$

$\mathcal{W=}Wh_{i}$ (1)

$e_{ij}=\alpha\left( Wh_{i}，Wh_{j} \right).$ (2)

To transform the input features into high-dimensional features (dimensional expansion) to achieve sufficient expressive power, each node undergoes a shared linear transformation as defined in Equation (1). The term $\mathcal{W}$ represents the high-dimensional features after transformation. The variable $e_{ij}$represents the correlation degree between nodes *i* and *j*. It can also be interpreted as the importance or contribution of node *j* to the central node *i*. In a conventional attention mechanism layer, once the correlation degrees between the central node and each neighboring node are obtained, a softmax function is applied to normalize these correlation coefficients, yielding the final attention weight scores. The equation is shown below, where $\kappa\in N_{i}$ denotes all neighboring nodes connected to node *i*:

${\alpha_{ij}={softmax}_{j} (e}_{ij})=\frac{exp(e_{ij})}{\sum_{\kappa\in N_{i}} exp(e_{ik})}$ (3)

In practical implementation, for each pair of nodes *i* and *j*, their features are concatenated into a

vector with a shape of (2×out_features). Then, a learned attention weight vector α∈(2×out_features,1) is applied to perform a linear transformation on the concatenated features, followed by the activation function LeakyReLU to retain elements greater than zero. By utilizing matrix transformations, computational efficiency is enhanced, thereby reducing time costs:

$$\alpha_{ij}=\frac{exp(LeakyRelu(\vec{\alpha}^{T}[(Wh_{i} || Wh_{j}]))}{\sum_{\kappa\in N_{i}} exp(LeakyRelu(\vec{\alpha}^{T}[(Wh_{i} || Wh_{k}]))} (4)$$

Following the above process, an attention score matrix, denoted as attention, is obtained to represent the *attention* coefficients between any two nodes. By overlaying a mask matrix of the same dimensions onto this matrix and incorporating information from the adjacency matrix, elements corresponding to zero entries in the adjacency matrix are mapped to a very small value to eliminate their influence. After passing through the softmax layer and dropout layer, the resulting matrix *attention′* is obtained. Finally, *attention′* is multiplied by $\mathcal{W}$ to obtain the updated state matrix:

$$h_{i}^{'}={attention}^{'}\cdot\mathcal{W} (5)$$

In a multi-head attention mechanism, different shared weights are used to obtain updated state matrices of various dimensions. These matrices are concatenated along the feature dimension to form a matrix with shape (20, heads×out_features), serving as the input to the output attention layer. This layer then reshapes the state matrix back to (20, out_features), effectively integrating the information gathered from different attention heads. This consolidated matrix is subsequently used as the input to the convolutional module.

**Hyperparameter Optimization and Selection**

In this study, Optuna was employed to optimize the hyperparameters of the graph neural network model. The suggest_categorical and suggest_int methods in Optuna were used to define the search space. The hyperparameters included learning rate (lr), L2 regularization coefficient (l2_lambda), batch size, hidden layer dimensions, dropout rate, number of attention heads, number of layers, activation function, the alpha parameter of LeakyReLU, and parameters specific to the Graph Attention Network (GAT) and Graph Convolutional Network (GCN). The objective task was designed under two-fold cross-validation (with the aim solely to find the optimal combination of hyperparameters rather than formal training) to ensure robust evaluation of model performance across different data splits. The metric used to evaluate optimization results was Mean Squared Error (MSE), and a total of 500 trials were conducted. Each trial represented a unique combination of hyperparameters, and the optimal model parameters were determined based on the trial with the lowest MSE. Tables S1 and S2 correspond to the hyperparameter combination space and the final hyperparameter values, respectively.

During these 500 optimization tasks, we conducted dynamic monitoring to track the progress of Optuna's experiments in real-time. For each trial, we calculated and reported the Spearman correlation coefficient mean, MSE mean, and Pearson correlation coefficient mean on a 2-fold cross-validation set. Violin plots (Fig.S3) of the Spearman and Pearson correlation coefficient optimization processes were generated to observe the stability and performance distribution of the model.

The violin plots (Fig.S3) reveal that both Pearson_mean and Spearman_mean have a median of approximately 0.83, indicating that the majority of trials achieved relatively high Pearson correlation coefficients. The interquartile range (IQR) between the upper and lower quartiles is narrow,

**Table S1 Hyperparameter Optimization Parameter Combination Space**

| Hyperparameter | | Range/Options |
| --- | --- | --- |
| Learning Rate (lr) | | [1e-5, 1e-4, 1e-3, 1e-2] |
| L2 Regularization (l2_lambda) | | [1e-6, 1e-5, 1e-4, 1e-3, 1e-2] |
| Batch Size | | [64, 128, 192, 256] |
| Hidden Dimensions | | [128, 256, 384, 512, 640, 768, 896, 1024, 1152, 1280, 1408, 1536, 1664, 1792, 1920, 2048] |
| Dropout Rate | | [0.1, 0.3, 0.5] |
| Number of Heads | | [1, 2, 3, 4, 5, 6, 7, 8] |
| Number of Layers | | [1, 2, 3] |
| Activation Function | | ['GELU', 'ReLU', 'LeakyReLU', 'Sigmoid', 'ELU', 'Tanh'] |
| Alpha (for LeakyReLU) | | [1e-3, 1e-2, 1e-1] |
| GAT Alpha | | [1e-3, 1e-2, 1e-1] |
| Convolution Layer | | ['GCNConv', 'GATConv', 'SAGEConv', 'GraphConv'] |
| Pooling Layer | ['TopKPooling', 'SAGPooling'] | |
| Global Pooling Layer | ['global_add_pool','global_mean_pool', 'global_max_pool'] | |

**Table S2 Final Selection of Hyperparameter**

| Name | Value |
| --- | --- |
| lr_values | 0.0001 |
| heads | 1 |
| hidden_dim | 1792 |
| layers | 3 |
| dropout | 0.1 |
| l2_lambda | 1e-05 |
| lr | 0.0001 |
| batch_size | 64 |
| alpha | 0.001 |
| Alpha | 0.001 |
| conv_layer | GCNConv |
| pool_layer | TopKPooling |
| global_pool_layer | global_mean_pool |
| activation | LeakyReLU |

suggesting that the performance of most trials is highly concentrated. Some outliers, with values below 0.6, represent underperforming trials, which primarily occurred during the initial stages of optimization. These outliers show significant divergence from the majority of trials.

Based on the optimization results, we can draw several conclusions: First, there is a high level of consistency. Most trials demonstrate consistently high performance on both Pearson and Spearman correlation coefficients, indicating that the majority of hyperparameter configurations perform well on the validation set. Second, a small number of underperforming trials are observed. Some outliers indicate that a few trials exhibited significantly lower performance compared to others. These trials likely employed unsuitable hyperparameter configurations, which should be avoided in subsequent experiments. Third, the model exhibits strong stability. Overall, the model demonstrates high performance stability across different hyperparameter configurations, with most trial metrics concentrated in the higher range.

In summary, employing the Optuna hyperparameter optimization strategy proves highly effective, enabling us to proceed with this model structure for further experiments.


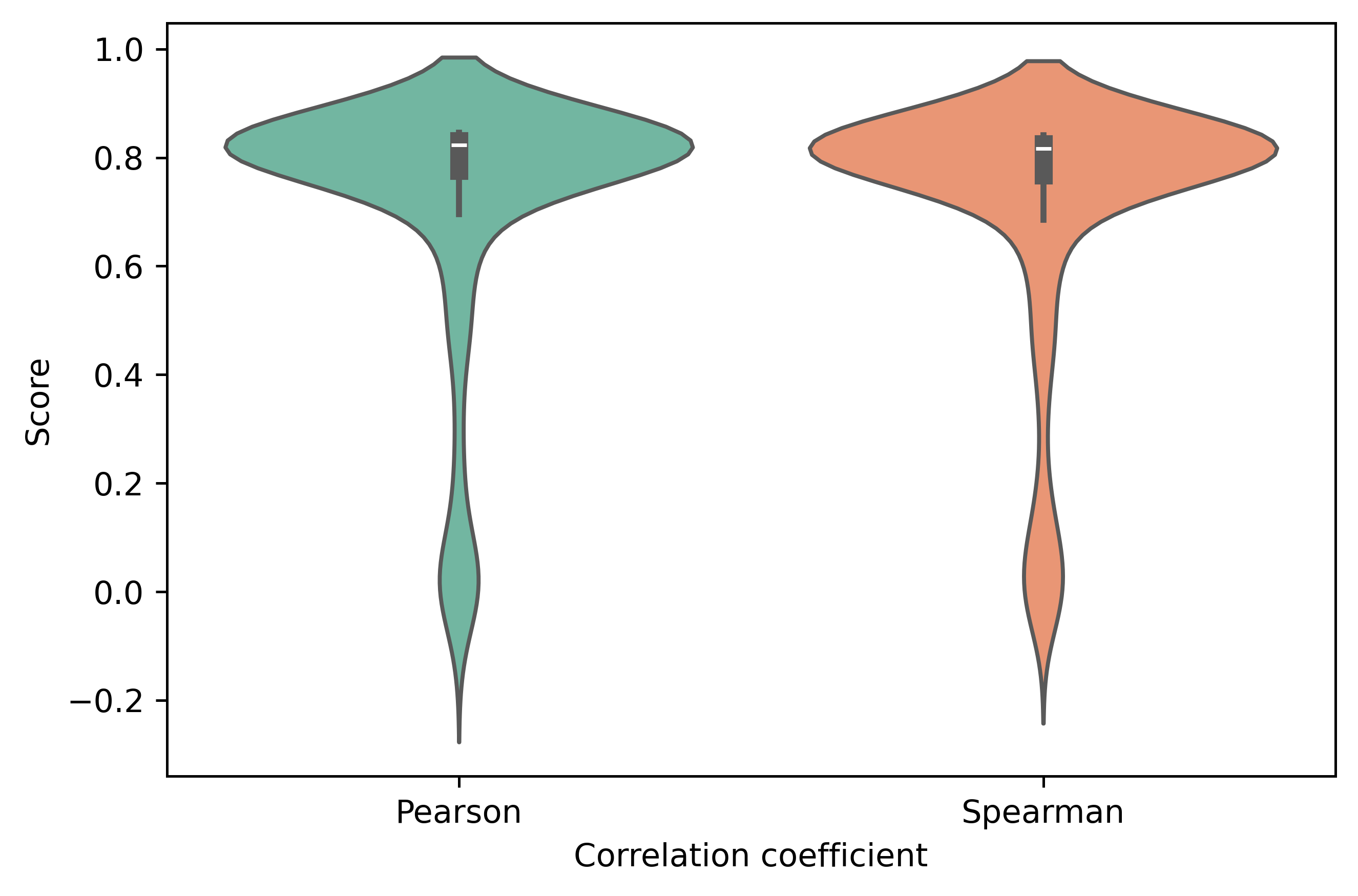


Fig.S3 Mean Spearman and Pearson correlation coefficients across two-fold validation trials.

**Selection of Graph Data Structures**

To ensure consistency of results, cross-validation was not applied in this experiment; instead, fixed training and testing (validation) sets were used. Mean Squared Error (MSE) was employed as the metric to evaluate each configuration, and the MSE for the eight structural configurations on the test set were plotted (Fig.2A). Finally, based on the experimental results, the best-performing configuration (C7) was selected as the data structure for subsequent experiments.

**Assessing Model Generalizability on Independent Datasets**

"Graph-CRISPR**^-^** " represents the graph model without pretraining. The prediction results are shown in Fig.2D, Table 3 and Table 4. However, considering dataset discrepancies, such as differences in experimental conditions and label computation heterogeneity, the baseline models were fine-tuned before being evaluated on specific test sets. To maintain methodological robustness, we additionally implemented a pretraining approach for the graph-based model, which is designated as Graph-CRISPR. The six datasets were randomly partitioned into 85% for training and 15% for testing (Table S3). A five-fold cross-validation was performed on the 85% training-validation set, and subsequently tested on the remaining test set.

Through a comparison of model performance, Graph-CRISPR exhibited high adaptability and effectively handled new datasets. Notably, after pretraining, the model obtained an average

**Table S3 Test Dataset Split**

| Dataset | Train-Val Set | Test set |
| --- | --- | --- |
| HL60 | 1765 | 312 |
| HELA | 6886 | 1215 |
| HCT116 | 3603 | 636 |
| WT | 47232 | 8341 |
| HF | 47150 | 8453 |
| ESP | 49823 | 8793 |

Spearman correlation coefficient of R=0.902 and an average Pearson correlation coefficient of r=0.918 on the six datasets, with consistent performance. Furthermore, we observed a noticeable decrease in accuracy across all models, including Graph-CRISPR, when applied to the three endogenous locus datasets (HCT116, HL60, and HeLa). However, following pretraining, Graph-CRISPR consistently outperformed the other models in both Spearman and Pearson correlation metrics (Table 3 and Table 4).

**Robustness Experiment**

Unlike the previously used CRISPR-Cas9 and PE systems, base editing enables precise genetic modifications by directly substituting specific bases. Moreover, BE encompasses multiple derivative editing systems. The unique mechanism of this system offers a robust platform for evaluating the generalizability of graph-based models across different editing systems. In CAELM, in addition to the basic BE4max system, two modified editing systems based on BE4max are also discussed: Anc-BE4max and hyA3A-BE4max.

Specifically, the original study selected a subset of 160 valid samples from the Be-set dataset and conducted experiments in two different cellular environments (Hek293T and HepG2) along with the two previously mentioned modified editing systems and the BE4max editing system. It should be noted that the three BE systems and the two cell lines would normally combine to generate six heterogeneous datasets. However, since the CAELM model was developed based on the experimental data generated by the basic BE4max editing system in the Hek293T cellular environment. Therefore, by combining the two cell lines and three editing systems, a total of other five datasets were generated. Although these datasets contain the same 160 samples, the differences in editing systems and cellular environments result in distinct label values, leading to experimental data with unique characteristics. In the Be-set literature, the authors adopted the optimal parameters of the CAELM model as initial conditions, followed by transfer learning on these five new datasets. Specifically, each dataset was divided into 85% for training and 15% for testing. The Pearson correlation coefficient was once again selected as the performance evaluation metric. To ensure experimental rigor and reproducibility, we adhered rigorously to the procedures outlined in the original experimental section when conducting the relevant experiments.

**Permutation Test Validation of Model Predictions**

To verify whether the predictive performance of the Graph-CRISPR model significantly exceeds random prediction on the WT and HCT116 datasets, we performed a permutation test. The specific procedure was as follows: First, we calculated the original Pearson/Spearman correlation coefficients based on the true labels and predicted labels of the test set. Subsequently, we randomly shuffled the true labels 10,000 times, recalculating the correlation coefficient between the shuffled labels and predicted labels after each shuffling to construct the null distribution. Finally, we computed the p-value by comparing the original correlation coefficient with the null distribution to assess statistical significance.

The permutation test results demonstrated that the Graph-CRISPR model's predictive performance significantly exceeded random prediction on both the WT and HCT116 datasets (p<0.05). Specifically, in the HCT116 dataset, the original correlation coefficient reached 0.94 (p=0.0001), with the true correlation coefficient located at the extreme right tail of the null distribution (Fig. S4A). Similarly, in the WT dataset, the original correlation coefficient was 0.92 (p=0.0001), also showing significant deviation from the center of the null distribution (Fig. S4B). The characteristics of the null distribution revealed that the correlation coefficients after label shuffling were concentrated around 0, while the significant deviation of the original correlation coefficient confirmed the non-random nature of the model's predictive capability. This analytical approach eliminated the influence of random chance and enhanced the statistical robustness of the conclusions.


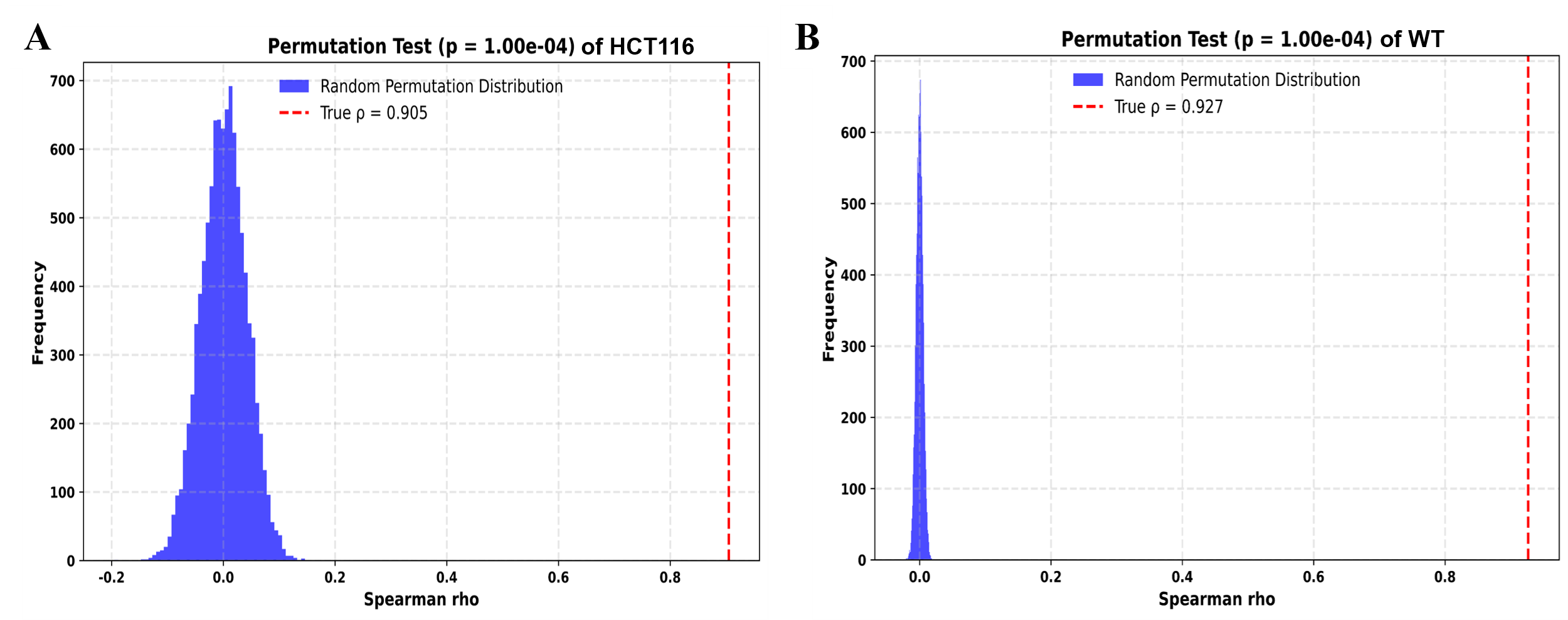


**Fig.S4 Visualization of Replacement Testing of (A) HCT116 and (B) WT.**

**Similarity Assessment Between Test and Training Sets and Data Filtering**

To systematically evaluate the potential risk of data leakage between the training and test sets, this study quantitatively characterized the sequence similarity distribution between the original training and test sets from multiple dimensions and conducted an in-depth analysis of sequence overlap features. For datasets of different scales, MMseqs2 (suitable for large-scale WT datasets) and SeqIO+Pairwise2 (suitable for small-scale HCT116 datasets) were employed to perform comprehensive sequence alignment analyses. The sequence overlap features were dissected through similarity distribution histograms, redundancy rate statistics, and alignment score distributions.

In the WT dataset, the alignment results based on MMseqs2 (Fig.S5A) revealed a significantly polarized distribution of similarity scores between test sequences and the training set: 98.9% of test sequences exhibited similarity scores below 0.2 (low-similarity region), while only 1.1% of sequences fell into the high-similarity region (0.8–1.0). Notably, the medium-similarity region (0.2–0.8) showed almost no sequence distribution (<0.1%). This distribution pattern aligns with the reported high RNA activity variability of the WT dataset [S1]. Quantitative statistics demonstrated that after threshold-based filtering (threshold = 0.9), the redundancy rate of the test set was only 2.24%. Furthermore, the mean and median values of all alignment scores were at low levels (specific values are shown in Table S4), further confirming significant functional divergence among sequences.

The similarity analysis of the HCT116 dataset (Fig.S5B) exhibited markedly different characteristics: due to the small scale of this dataset and the inherent clustering effect of sgRNA design [S2], 96% of test sequences had similarity scores concentrated in the 0.6–0.8 range (high internal consistency region). Although the medium-similarity region (0.4–0.6) contained only 150 sequences (~15% of the total), the high-similarity region (0.8–1.0) showed almost no sequence distribution. This distribution pattern is consistent with previous studies reporting high homology in HCT116 sgRNA design. Statistical analysis revealed that after threshold-based filtering (threshold = 0.9), the redundancy rate was 2.52%, with an average alignment score of 11.9, indicating that despite design-related correlations, the sequences maintained sufficient distinguishability to avoid functional overlap.

Comparative analysis demonstrated that the WT dataset, owing to its inherent sequence diversity, exhibited minimal risk of sequence overlap, while the HCT116 dataset, despite showing design-related correlations, could effectively eliminate potential interfering sequences through strict similarity threshold control. Across both scenarios, the clear boundaries of similarity distribution histograms, significant reduction in redundancy rates, and low alignment score distributions collectively formed a threefold evidence chain for data leakage assessment. These findings validated the reliability of the original model performance evaluation.


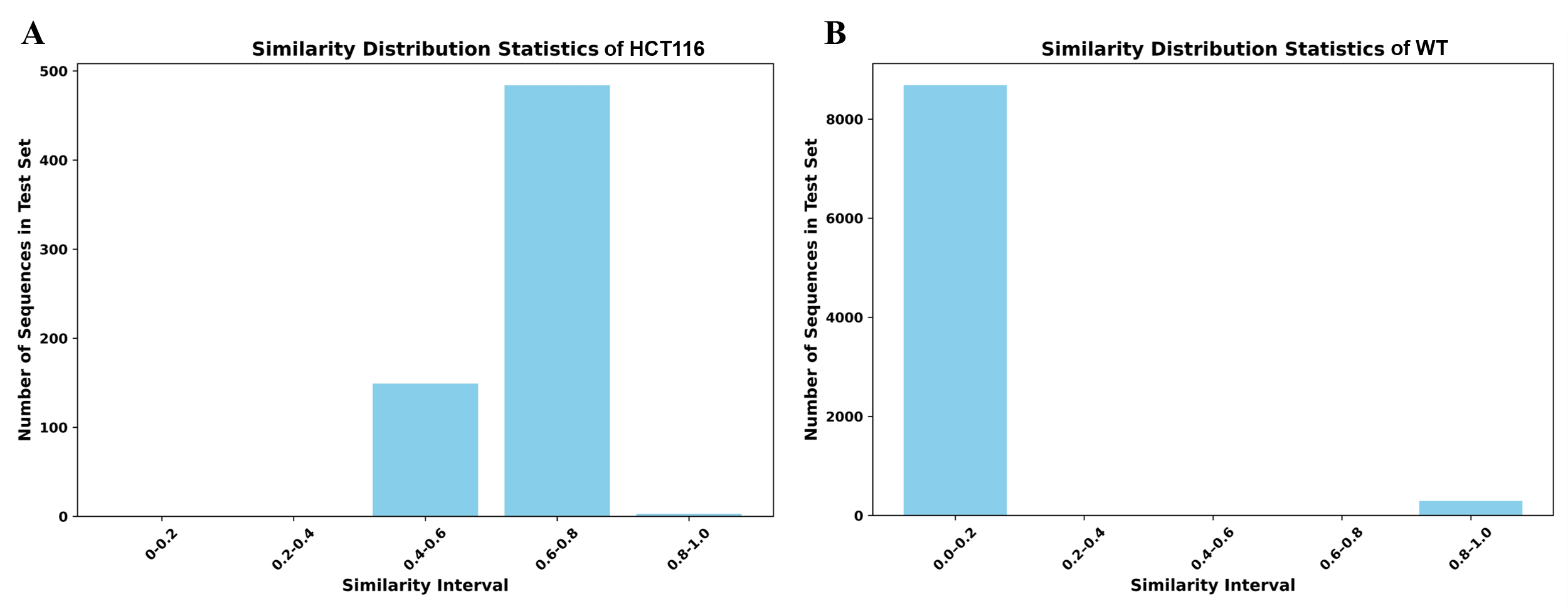


**Fig.S5 Similarity distribution histogram (A) of HCT116 dataset, (B) of WT dataset (threshold = 0.9).**

**Table S4 Statistical analysis for the WT and HCT116 (threshold = 0.9).**

|  | **Mean Similarity (%)** | **Median Similarity (%)** | **Max**  **Similarity (%)** | **Min**  **Similarity (%)** |
| --- | --- | --- | --- | --- |
| **WT** | 3.2 | 0 | 100 | 0 |
| **HCT116** | 11.9 | 12 | 19 | 6 |

**Predictive Performance on Filtered Test Sets**

We constructed four test datasets through sequence similarity comparison: for the small-scale HCT116 dataset, we used the Biopython tool to screen sequences with similarity thresholds of 0.9 and 0.8, respectively, resulting in HCT116_test1 with 16 highly similar sequences removed and HCT116_test2 with 107 highly similar sequences removed; for the large-scale WT dataset, we switched to the MMseqs2 tool and obtained 8,776 sequences with a similarity threshold of 0.9 (WT_testset1) and 8,686 sequences with a similarity threshold of 0.8 (WT_testset2) from a randomly selected set of 8,978 sequences.

Fig.S6 comprehensively presents the performance of the Graph-CRISPR model across the four test datasets (HCT116_test1, HCT116_test2, WT_test1, and WT_test2) for both the HCT116 and WT datasets. The model performance remains highly consistent with the original results shown in Fig. 2D, with the fluctuation in Spearman correlation coefficients being less than 0.026, thereby validating the effectiveness of the data filtering strategy—eliminating potential data leakage risks while retaining sufficient sequence diversity to assess the model’s generalization capability.

Specifically, in the original HCT116 test dataset (Fig.2D), the Pearson correlation coefficient of the Graph-CRISPR model was 0.92; after sequence filtering, the Spearman correlation coefficients for the two filtered test datasets (HCT116_test1 and HCT116_test2) increased to 0.94 and 0.941, respectively. For the two filtered WT test datasets (WT_test1 and WT_test2), the Pearson correlation coefficient of the model improved from the original 0.914 to 0.918 and 0.921, respectively (Fig.S6).

On the filtered test sets with highly similar sequences removed, the model’s predictive results demonstrate remarkable robustness, maintaining consistently high performance on both the WT and HCT116 datasets—both Pearson and Spearman correlation coefficients remain at elevated levels, with particularly notable improvements observed in the HCT116 dataset. Furthermore, the model exhibits low sensitivity to changes in similarity thresholds. These findings collectively validate the model’s generalization capability. The results not only confirm the reliability of the original model performance evaluation but also highlight the critical role of data preprocessing. By implementing strict similarity control (WT sequence retention rate: 96.75%/97.76%; HCT116: 83.2%/97.5%), this strategy significantly enhances prediction robustness while minimally compromising data representativeness. Future research could explore dynamic threshold strategies to balance data utilization efficiency and leakage risk mitigation.


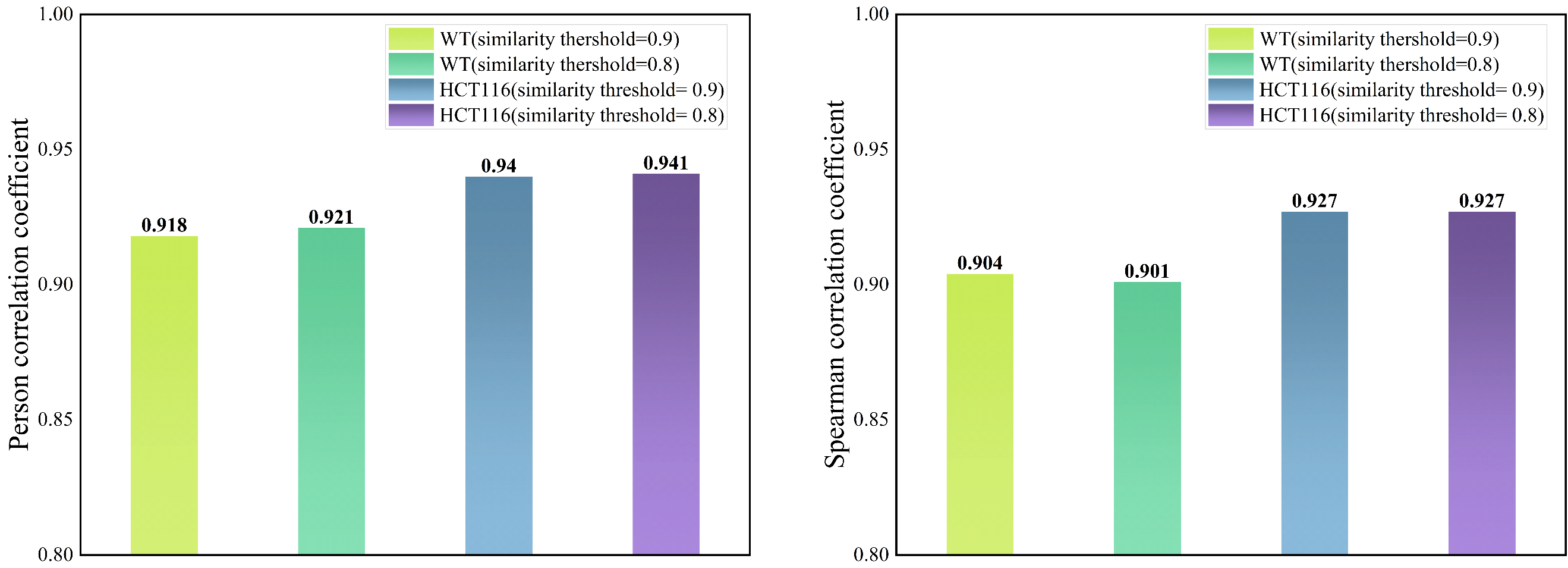


**Fig.S6 Sequence analysis experimental test result**

**Interpretability of Graph-CRISPR based on heatmap of attention**

Deep learning models generally lack credibility due to their "black box" mechanism, with most being oriented towards the goal of predictive accuracy, and lacking further contemplation on the models themselves. Therefore, we attempted to enhance the interpretability of Graph-CRISPR by outputting intermediate process matrices, ultimately selecting the GAT graph attention neural network module for further analysis. The graph attention mechanism itself is an application of attention methodology transferred to the field of graph neural networks, based on the attention scoring mechanism of the graph attention mechanism, which provides some references and insights for interpreting deep learning models. Specifically, we chose the Graph-CRISPR model's training dataset, Kim's Train, as the baseline dataset because it has been widely used in the development of several targeted models in recent years, demonstrating its universality and credibility.

We first retrained the model on Kim's Train. During this training process, we collected the attention score matrices output by GAT by adding attention score recovery instructions in the GAT module and visualized this result using attention heatmaps. Kim's Trains, as the development dataset for graph models, contains a total of 12,832 data points. Since the graph data used in this paper consists of 20 nodes, we obtained 12,832 attention score matrices of size 20×20. Due to the large amount of data, we could not visualize all the attention heatmaps; therefore, we randomly selected 4 groups of node attention score heatmaps using random seeds, with each group containing 9 graphs of node attention scores, as shown in Fig.S7.

The X-axis represents the target position, which indicates where the model focuses when generating outputs, while the Y-axis denotes the source position. The color bar on the right side of the figure indicates the intensity of the attention scores, with colors ranging from light yellow to dark red,


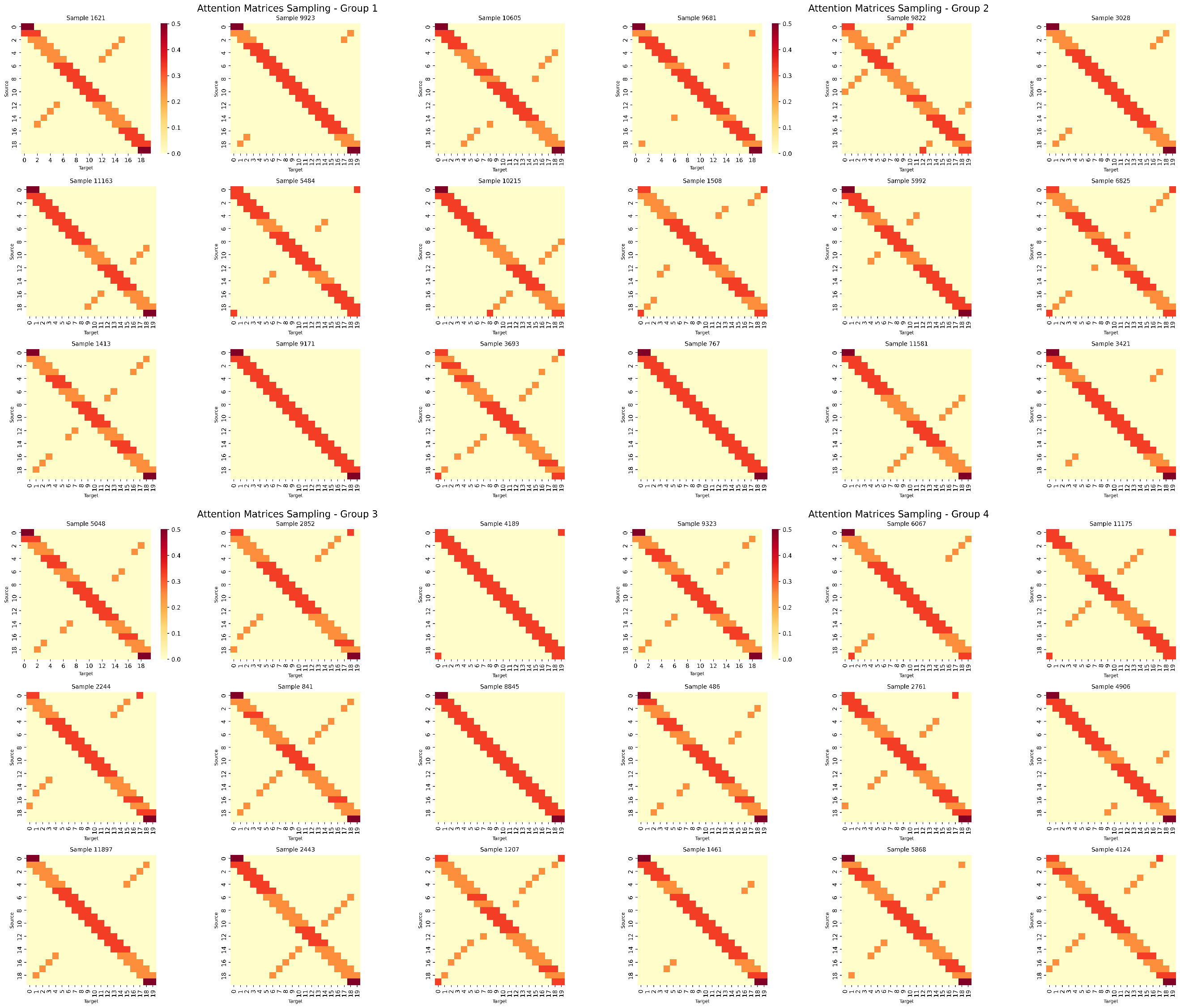
**Fig.S7 Heatmap of attention scores under random sampling.**

corresponding to scores from low to high. The deeper the color, the higher the attention score, meaning the model is more reliant on the corresponding position when generating outputs. Firstly, in most graphs, it can be observed that points on the diagonal are colored darker, indicating a higher attention score between adjacent positions in the sequence. This is expected, as adjacent sgRNA sequences are likely to be functionally related. Additionally, aside from the diagonal, some subplots also contain high attention score areas that are non-diagonal. These are attributed to the influence of the edges of secondary structures, further emphasizing the importance of incorporating this critical feature into our model. By capturing both adjacent nodes and long-range dependencies, we can effectively support the updating of central node features, which in turn enables the model to achieve more precise predictive performance. However, merely exhibiting node heatmaps through sampling does not comprehensively reveal the overall analytical results. To gain a deeper understanding of the attention score matrix, we conducted analyses in the following aspects.

In graph data, each node plays a dual role in the feature update process of Graph Neural Networks (GNNs): on one hand, it serves as a target node (central node) receiving information from other nodes; on the other hand, it acts as a source node (contributing node) providing information to other target nodes. Based on this dual role, we conducted a detailed positional analysis and generated three importance ranking charts (Fig.4) to quantify the significance of each node under different circumstances. (1) Target Node Importance (Left): This chart calculates the total attention score received by each node when acting as a target node. It reflects the importance of each node as an information receiver during the feature update process. (2) Source Node Importance (Middle): This chart calculates the total attention score emitted by each node when acting as a source node. It reveals the influence of each node as an information contributor in the feature update process. (3) Comprehensive Node Importance (Right): This metric highlights hub nodes that are both important in themselves and highly regarded by other positional nodes, thereby identifying nodes that play a critical role in the network. Through these three charts, we are able to comprehensively assess the role and importance of each node in the Graph Neural Network. In particular, the comprehensive node importance chart not only considers the information receiving and sending capabilities of the nodes themselves but also takes into account the overall influence of the nodes in the network, thus helping us identify strategically significant hub nodes in graph data. These analytical results are crucial for understanding and optimizing the performance of Graph Neural Networks, as well as for designing more effective graph structures in practical applications.

The analysis results indicate that in the assessment of target node importance, the 2nd and 19th bases exhibit significant importance characteristics, while the source node importance analysis further reveals the critical roles of the 2nd, 3rd, and 18th, 19th bases. A comprehensive evaluation shows that the 19th and 2nd bases rank as the top two in overall importance, a finding that is highly consistent with existing research: numerous studies on the gene editing efficiency of the CRISPR-Cas9 system have confirmed that there is a noticeable sequence preference for the 2nd and 19th bases of sgRNA, which have a decisive impact on the stability of the sgRNA-Cas9 complex and the efficiency of DNA cleavage [S3-S5].

Our analysis results (Table S5) provide a detailed record of the importance indicators for each base position (including target importance, source importance, impact score), and the rank according to their impact score. Notably, the correlation coefficient of target-source node significance is as high as 0.92 (Pearson correlation coefficient). This significant result indicates a high degree of synergy in the roles of bases as information receivers (targets) and providers (sources), suggesting that key base positions in sgRNA (such as positions 2 and 19) exhibit dual functional characteristics within the information transmission network: simultaneously serving as important information receiving nodes (high target significance) and as critical information sending nodes (high source significance). Furthermore, the high correlation also demonstrates the global coherence of the sgRNA sequence, implying a strong synergistic interaction among the bases of sgRNA, where editing efficiency may be jointly regulated by multiple sites rather than determined by isolated sites.

We conducted a comprehensive assessment of the top 10 ranked nucleotides in terms of importance, which are the 2nd to 6th positions at the 5' end of sgRNA and the 15th to 19th positions at the 3' end. The key positions identified by the model are highly consistent with multiple published literature and biological experimental results. First of all, the 5' end of sgRNA (especially positions 2 to 6) is known as the 'seed sequence'. From the perspective of the mechanism of action of gene editing, this region directly affects the initial binding stability of the Cas9/sgRNA complex to the target DNA. Near the PAM sequence region, specifically positions 15 to 19 of sgRNA, the HNH nuclease domain of Cas9 is closely related to the DNA unwinding process mediated by these positions, which similarly has a significant impact on the gene-editing effect. Multiple studies have confirmed that the editing efficiency of sgRNA is closely related to the seed sequence at the 5' end and the sequence near the PAM region at the 3' end [S6] , which even jointly determines the editing effect [S7]. These experimental findings further validate the reliability of our Graph-CRISPR model predictions. If we view the top-ranked important positions in sgRNA as 'hub bases', due to the fact that these hub bases not only affect other positions but are also influenced by feedback from other positions, we can prioritize attention and optimization of these hub bases during sgRNA design. Such a strategy is expected to further enhance the editing efficiency of sgRNA. Through this detailed analysis and optimization, we can design efficient and highly specific sgRNAs, providing a more solid foundation for the application of gene editing technology.

**Table S5 Analysis for the Importance of Position**

| No | target importance | source importance | impact score | Rank1 | Average Degree | Rank2 |
| --- | --- | --- | --- | --- | --- | --- |
| 1 | 0.8533 | 0.7068 | **0.7801** | **18** | **2.75** | **18** |
| 2 | 0.8422 | 0.7086 | **0.7754** | **1** | **2.70** | **2** |
| 3 | 0.7107 | 0.7082 | **0.7095** | **2** | **2.50** | **9** |
| 4 | 0.7114 | 0.707 | 0.7092 | 17 | 2.45 | 12 |
| 5 | 0.7099 | 0.7061 | 0.7080 | 15 | 2.35 | 14 |
| 6 | 0.7075 | 0.7067 | 0.7071 | 3 | 2.30 | 0 |
| 7 | 0.7087 | 0.7048 | 0.7068 | 14 | 2.30 | 5 |
| 8 | 0.7088 | 0.7043 | 0.7066 | 5 | 2.30 | 8 |
| 9 | 0.7064 | 0.7055 | 0.7060 | 4 | 2.30 | 10 |
| 10 | 0.7046 | 0.7053 | 0.7050 | 16 | 2.20 | 1 |
| 11 | 0.7065 | 0.7013 | 0.7039 | 13 | 2.20 | 17 |
| 12 | 0.7032 | 0.6993 | 0.7013 | 6 | 2.15 | 3 |
| 13 | 0.697 | 0.6941 | 0.6956 | 12 | 2.15 | 4 |
| 14 | 0.6928 | 0.6914 | 0.6921 | 7 | 2.15 | 7 |
| 15 | 0.683 | 0.6855 | 0.6843 | 11 | 2.15 | 13 |
| 16 | 0.6787 | 0.683 | 0.6809 | 8 | 2.05 | 6 |
| 17 | 0.6776 | 0.6806 | 0.6791 | 10 | 2.00 | 15 |
| 18 | 0.6772 | 0.6799 | 0.6786 | 9 | 1.95 | 19 |
| 19 | 0.4328 | 0.5808 | 0.5068 | 0 | 1.90 | 11 |
| 20 | 0.4143 | 0.5676 | 0.4910 | 19 | 1.85 | 16 |
|  | **Target-Source importance correlation**  **0.9198** | | | |  |  |

**GNNExplainer based interpretation**

To systematically investigate the decision-making mechanism of graph neural networks and enhance model credibility, we further employed GNNExplainer to conduct fine-grained analysis of the graph neural network's decision process. This method generates vertex-level explanatory subgraphs to quantify the structural importance of nodes in regression prediction. Specifically, it first identifies the key connection patterns of each vertex during updates, then extracts the local structures that most significantly influence the prediction target through subgraph optimization algorithms, and finally uses vertex degree (the actual number of connected neighboring vertices) as the metric for importance quantification—vertices not included in the subgraph are assigned a degree value of 0, while those included are assigned a degree value equal to their actual number of connections in the subgraph. By calculating the average degree of each vertex across multiple subgraphs, we assessed its global importance across samples.

Analysis of the Kim's Train dataset (where each sgRNA sequence was structured as a graph with vertices numbered 0-19) demonstrated that among the top three ranked vertices identified by both methods, two vertices (18 and 2) showed agreement. This finding, with an occurrence probability of only 4.47% (p < 0.05), is statistically significant and cannot be attributed to random chance. Notably, vertex 18 consistently ranked first across both methods, while vertex 2 was identified as the 3rd and 2nd most important vertex in the two methods respectively. This cross-method validation confirms that graph neural networks can simultaneously capture both the global attention distribution patterns of RNA sequences and local subgraph structural features, thereby enabling precise identification of key binding sites. The consistency of important nodes identified at different scales by the two methods not only validates the robustness of the model's predictive performance, but also reveals the decision-making mechanism whereby graph neural networks achieve accurate predictions through the integration of local structural features (from GNNExplainer) and global topological information (from attention mechanisms).

# Supplementary Figures


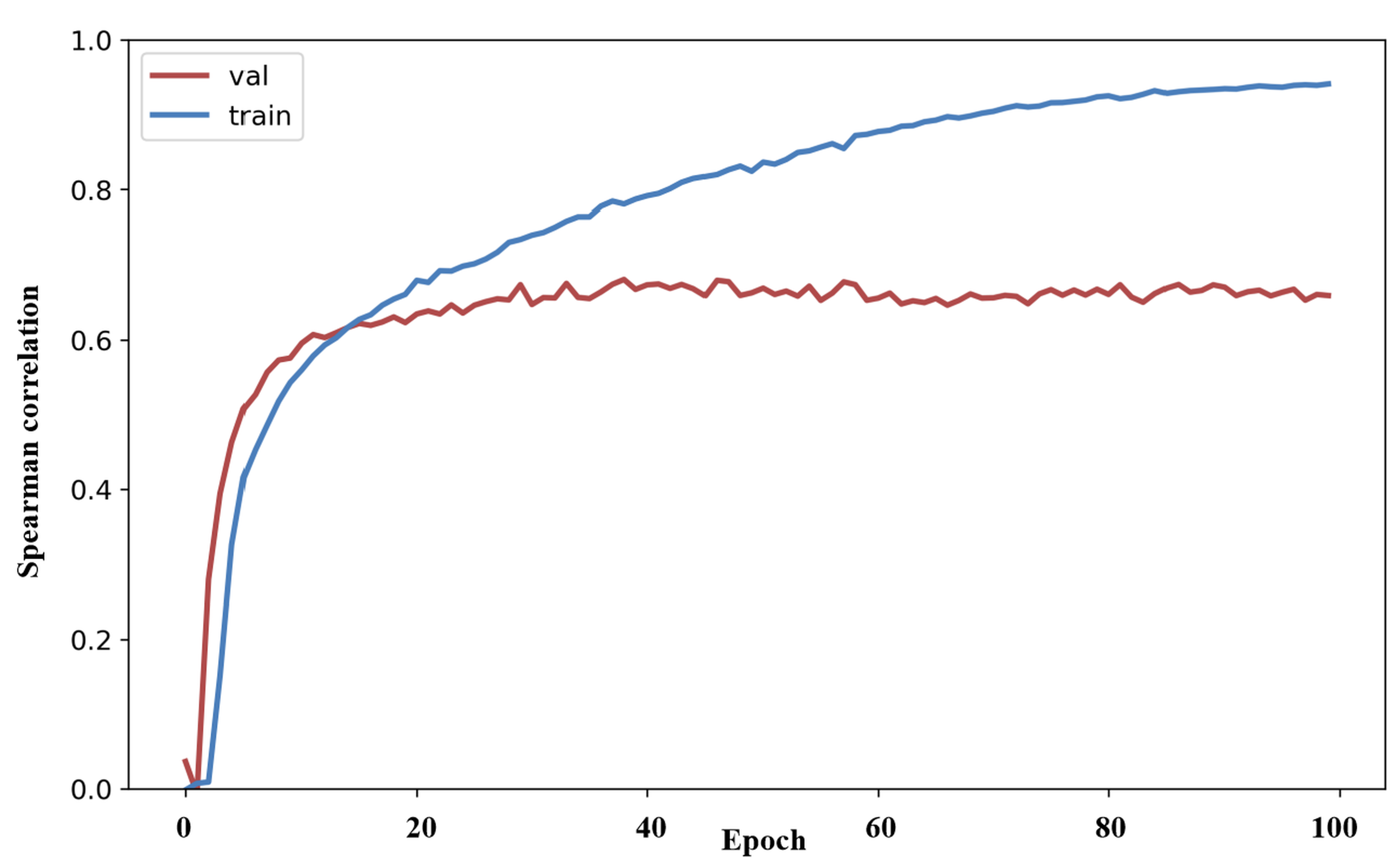


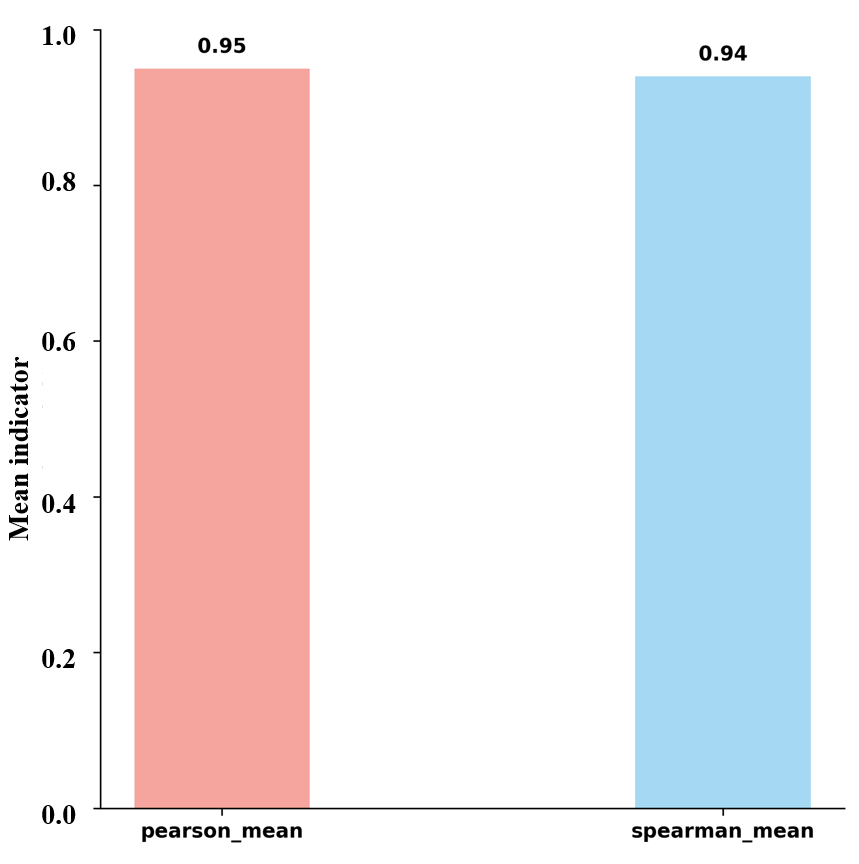
**Fig.S8 Spearman correlation curves of valid and training sets for MXfold2+RNA-FM+20bp combination.**

**Fig.S9 Mean Spearman and Pearson correlation coefficients in two-fold cross-validation**


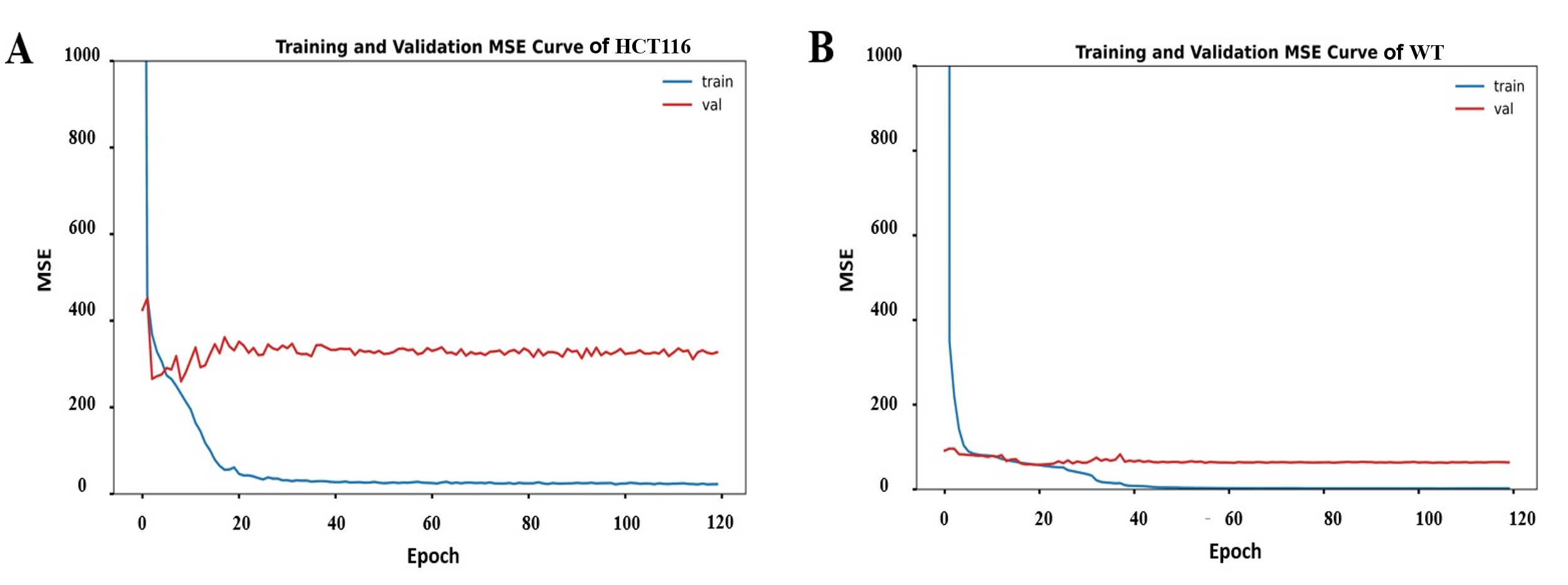


**Fig.S10 Training curves for retrained HCT116 (A) and WT (B) datasets.**

**Reference**

1. Wang, D., Zhang, C., Wang, B. et al. (2019). Optimized CRISPR guide RNA design for two high-fidelity Cas9 variants by deep learning. Nature communications, 10(1), 4284.
2. Hart, T., Chandrashekhar, M., Aregger, M. et al. (2015). High-resolution CRISPR screens reveal fitness genes and genotype-specific cancer liabilities. Cell, 163(6), 1515–1526.
3. Doench JG, Hartenian E, Graham DB et al. Rational design of highly active sgRNAs for CRISPR-Cas9-mediated gene inactivation, Nat Biotechnol 2014;32:1262-1267.
4. Wang T, Wei JJ, Sabatini DM et al. Genetic screens in human cells using the CRISPR-Cas9 system, Science 2014;343:80-84.
5. Xu H, Xiao T, Chen CH et al. Sequence determinants of improved CRISPR sgRNA design, Genome Res 2015;25:1147-1157.
6. Liu X, Yang J, Song Y et al. Effects of sgRNA length and number on gene editing efficiency and predicted mutations generated in rice, The Crop Journal 2021.
7. Yuan T, Wu L, Li S et al. Deep learning models incorporating endogenous factors beyond DNA sequences improve the prediction accuracy of base editing outcomes, Cell Discov 2024;10:20.
