# Supplementary figures and images for: Graph-CRISPR: a gene editing efficiency prediction model based on graph neural network with integrated sequence and secondary structure feature extraction
Source: Brief Bioinform. 2025 Aug 15;26(4):bbaf410. doi: 10.1093/bib/bbaf410 (PMC12354951; doi:10.1093/bib/bbaf410)

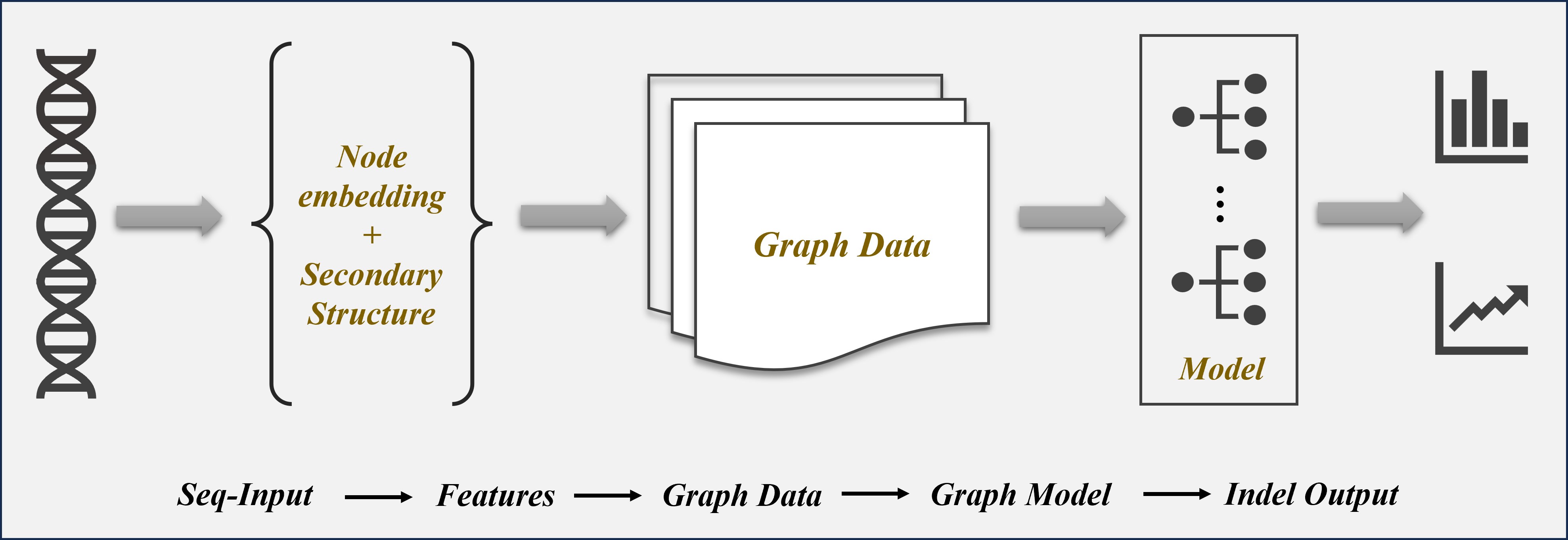

Supplement: Fig_S1_bbaf410 [file fig_s1_bbaf410.jpeg]

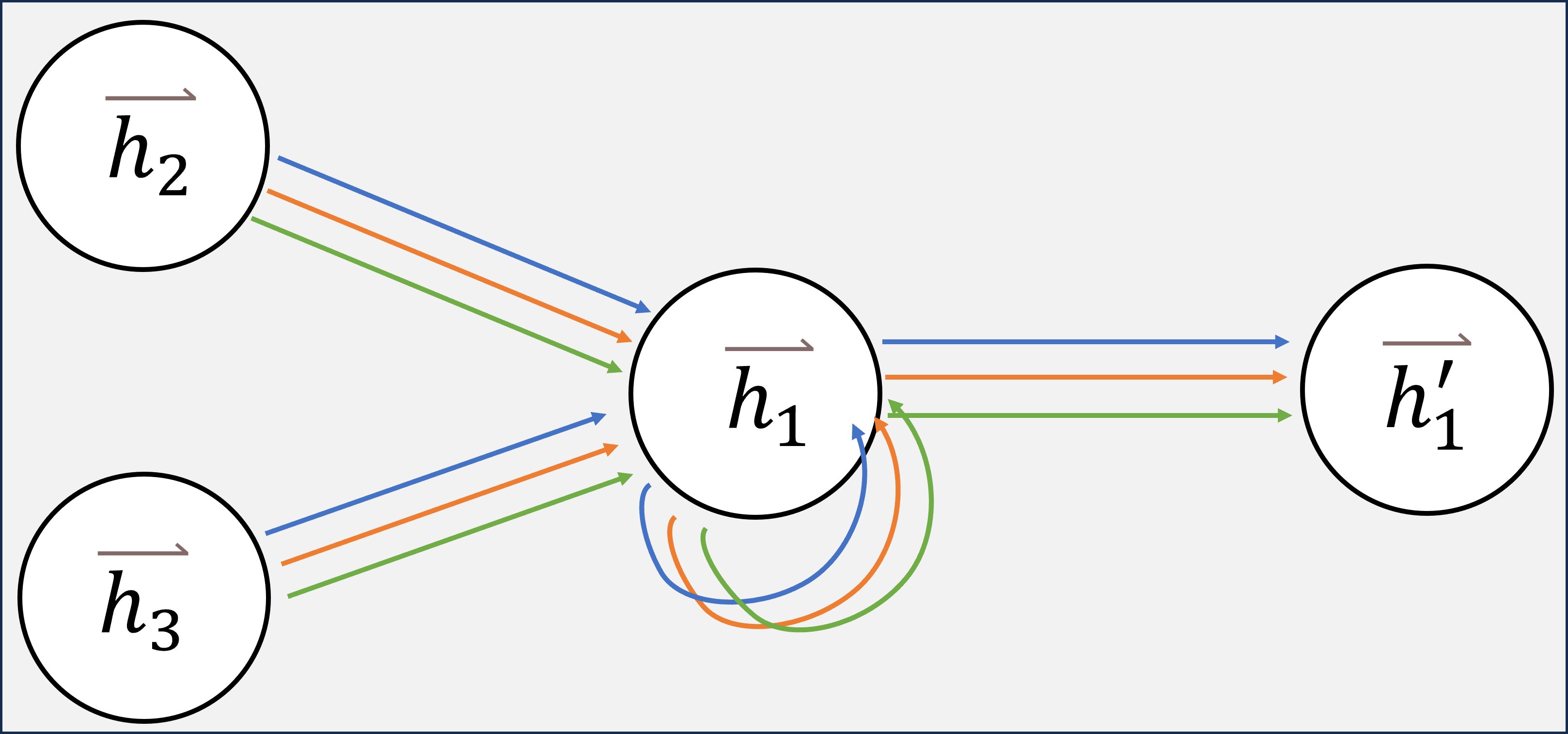

Supplement: Fig_S2_bbaf410 [file fig_s2_bbaf410.jpeg]

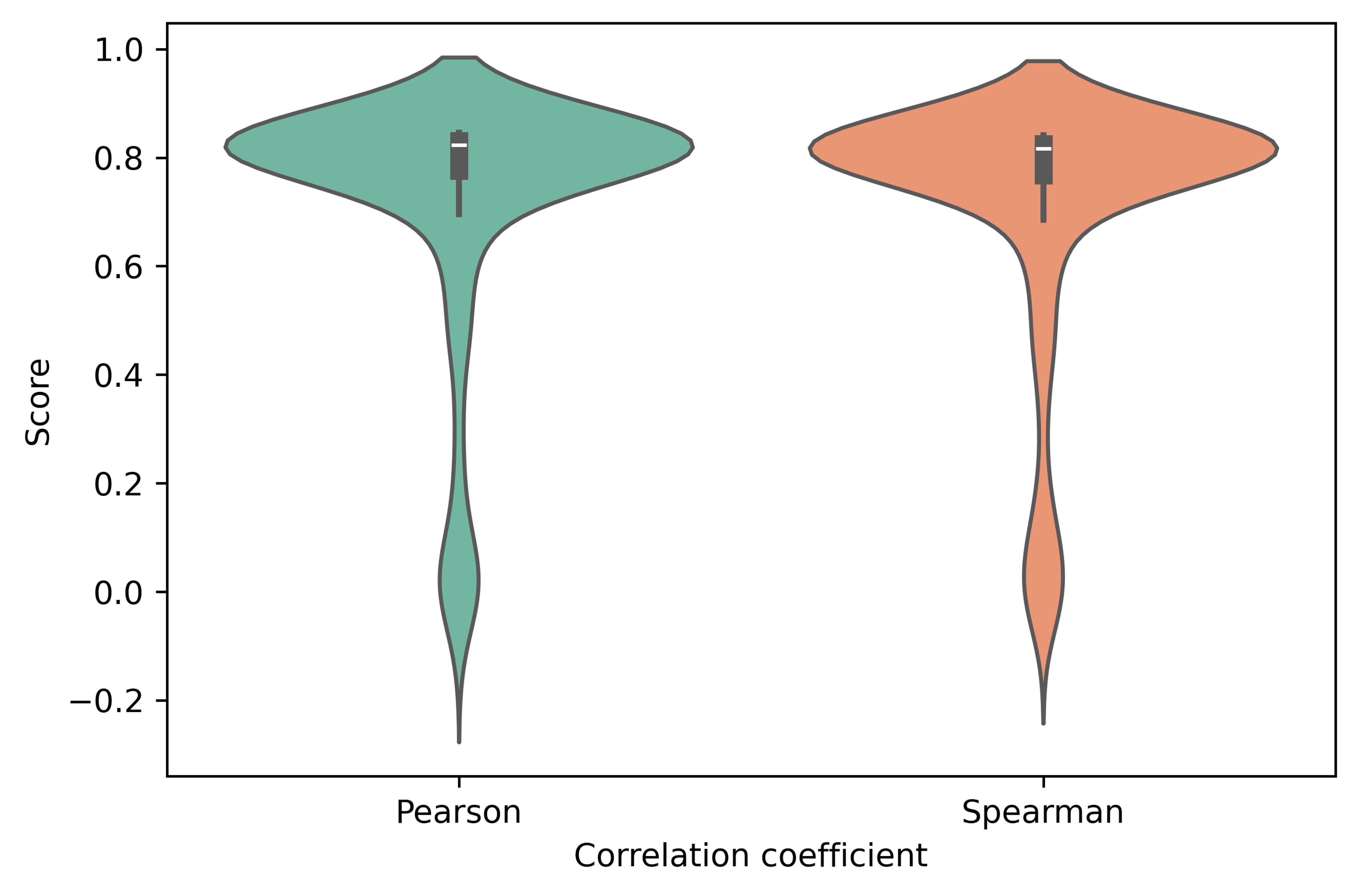

Supplement: Fig_S3_bbaf410 [file fig_s3_bbaf410.jpeg]

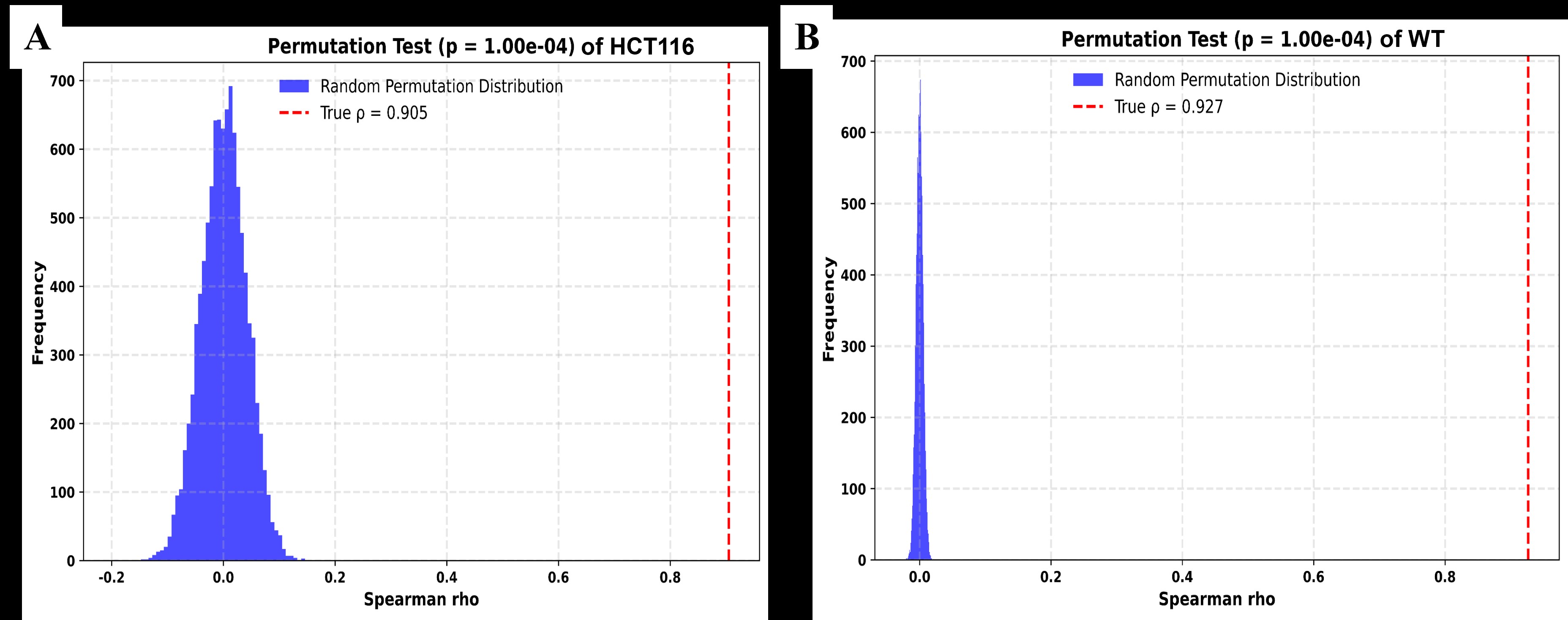

Supplement: Fig_S4_bbaf410 [file fig_s4_bbaf410.jpeg]

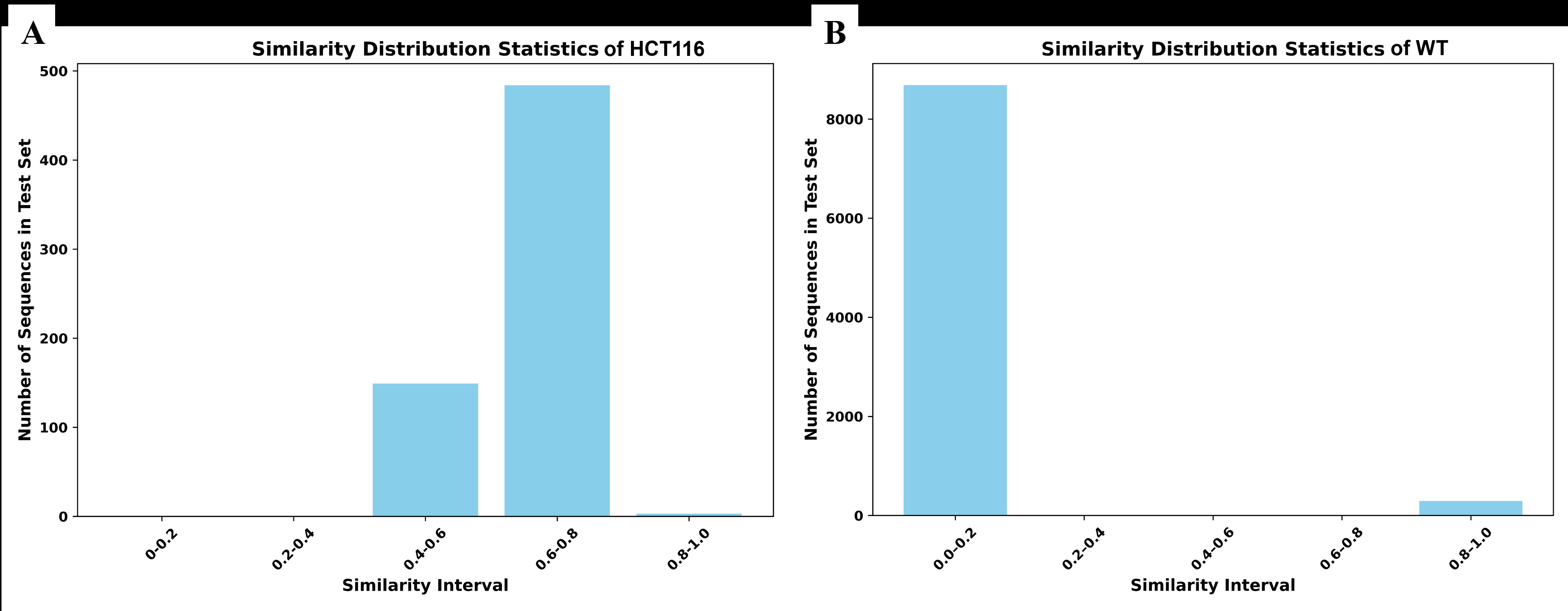

Supplement: Fig_S5_bbaf410 [file fig_s5_bbaf410.jpeg]

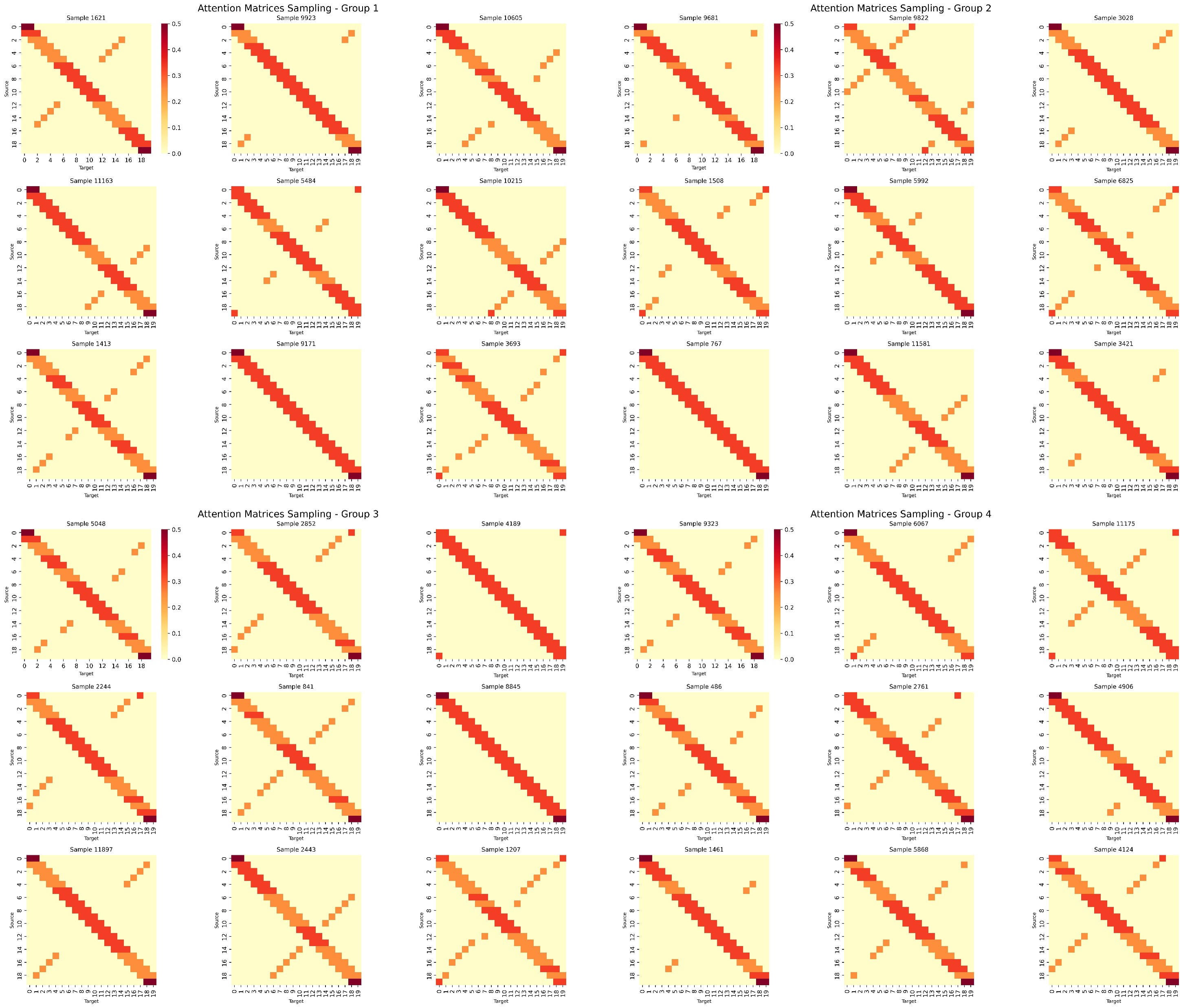

Supplement: Fig_S7_bbaf410 [file fig_s7_bbaf410.jpeg]

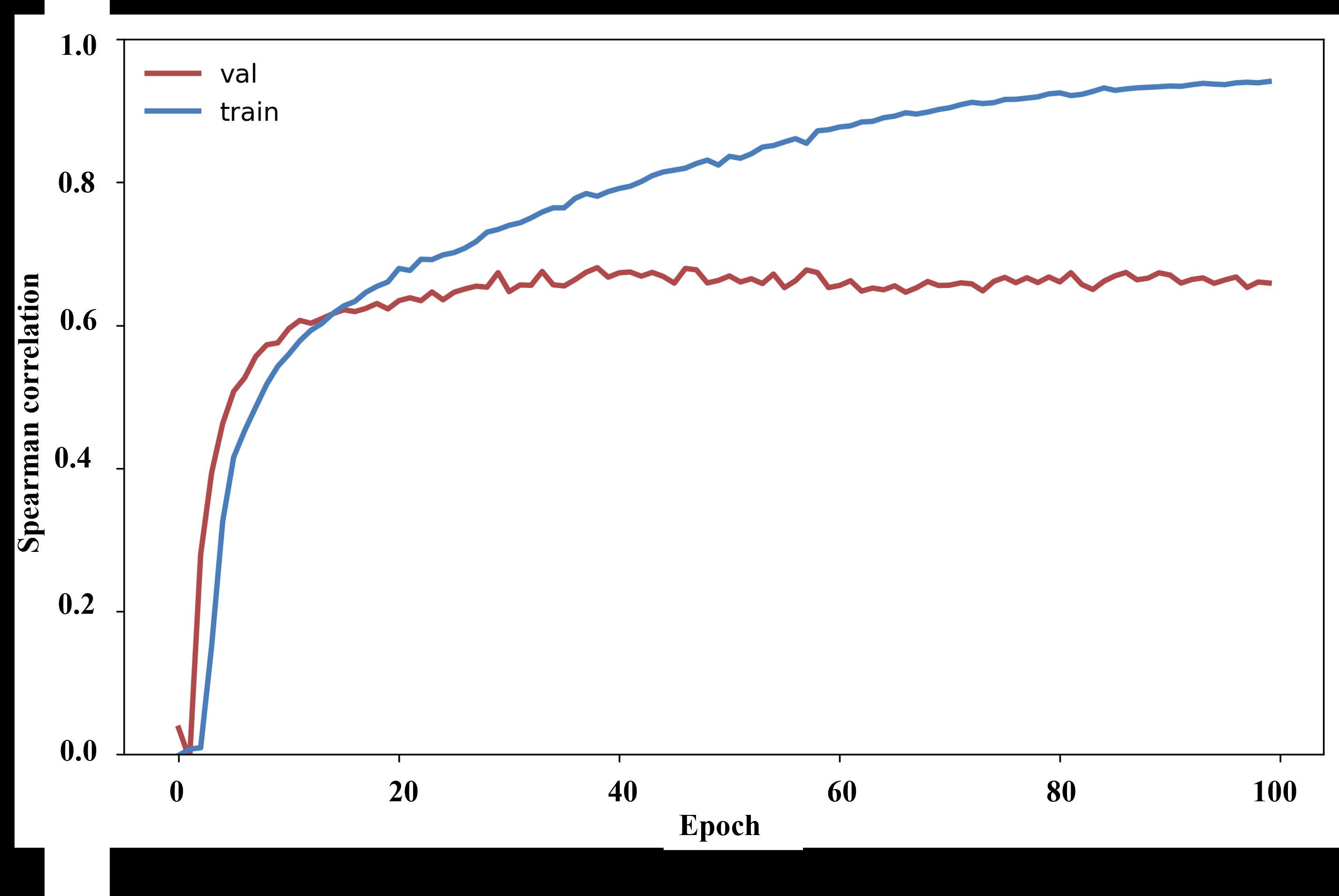

Supplement: Fig_S8_bbaf410 [file fig_s8_bbaf410.jpeg]

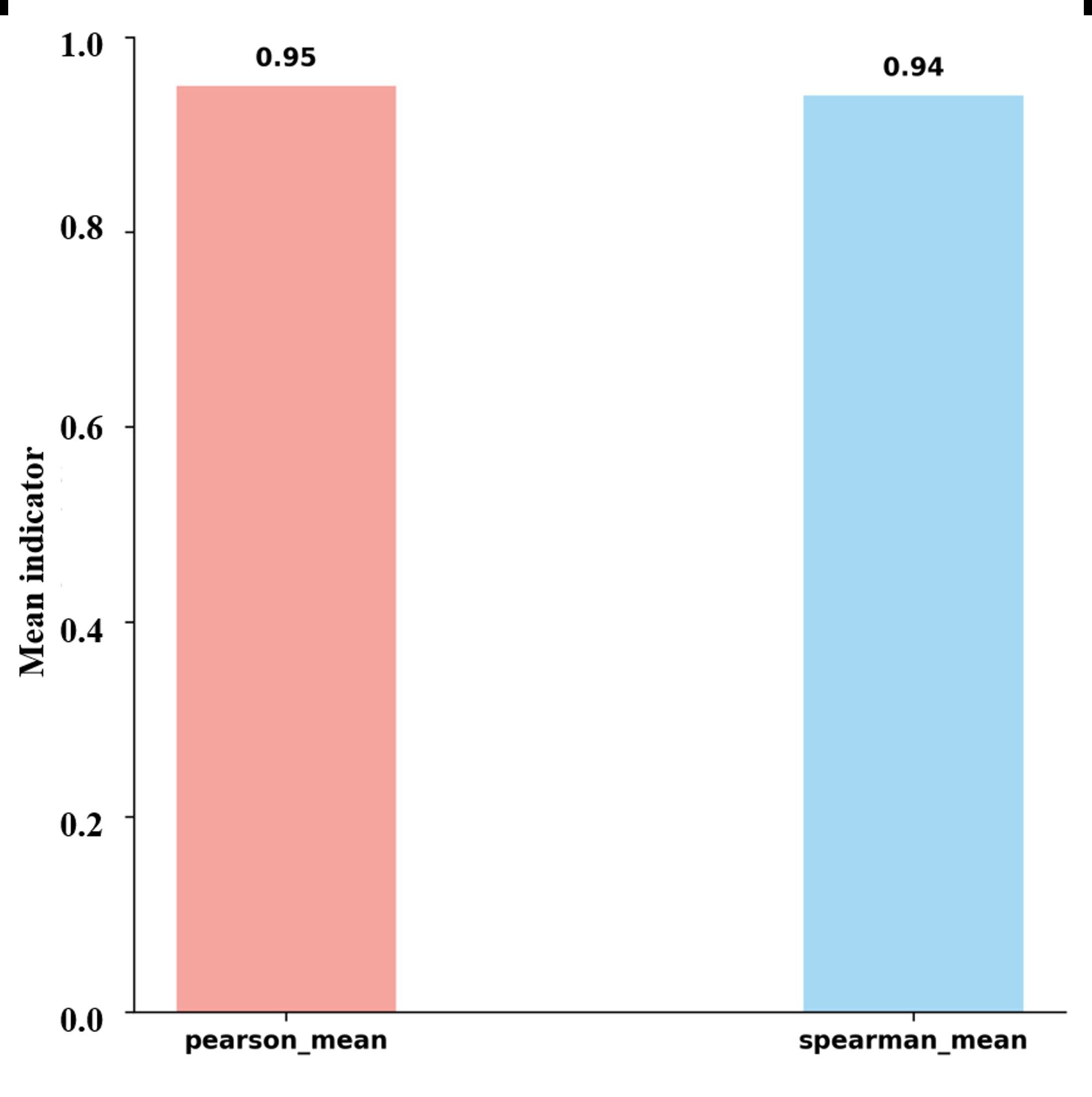

Supplement: Fig_S9_bbaf410 [file fig_s9_bbaf410.jpeg]

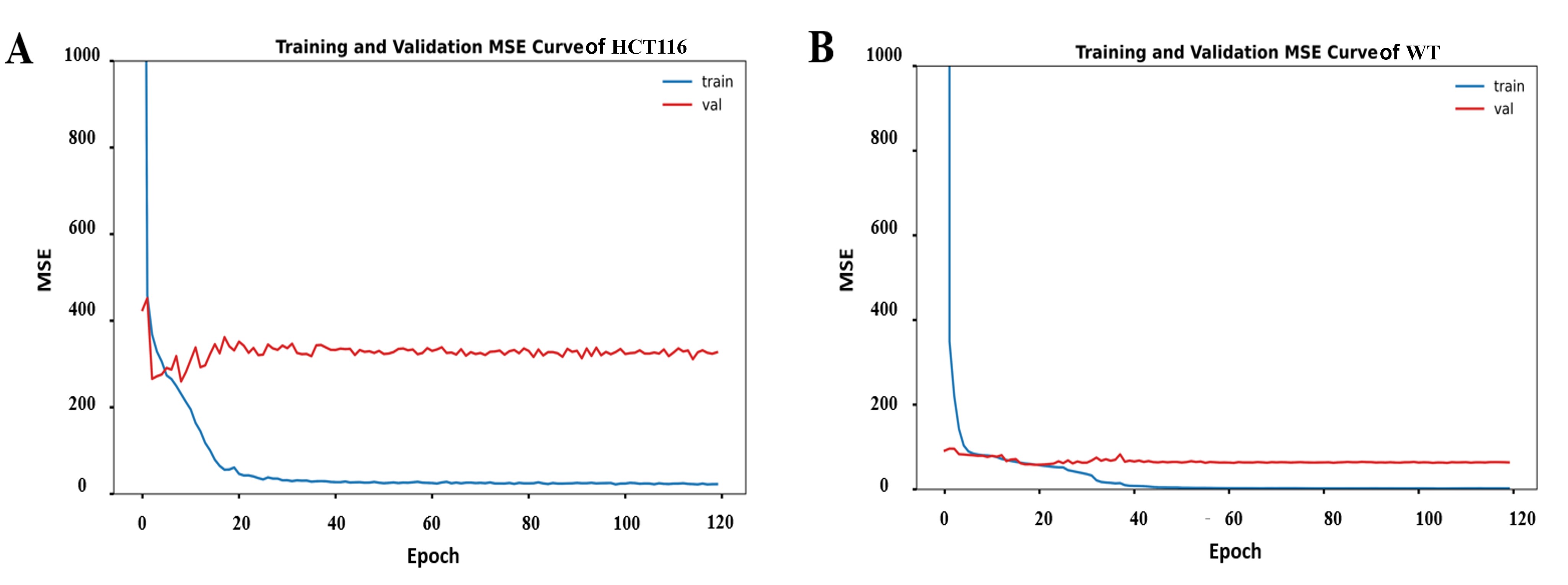

Supplement: Fig_S10_bbaf410 [file fig_s10_bbaf410.jpeg]
