# Supplementary material for: Graph-CRISPR: a gene editing efficiency prediction model based on graph neural network with integrated sequence and secondary structure feature extraction
Source: Brief Bioinform. 2025 Aug 15;26(4):bbaf410. doi: 10.1093/bib/bbaf410 (PMC12354951; doi:10.1093/bib/bbaf410)
Supplement: Table_S1_Hyperparameter_Optimization_Parameter_Combination_Space_bbaf410 [file table_s1_hyperparameter_optimization_parameter_combination_space_bbaf410.docx]

**Table S1 Hyperparameter Optimization Parameter Combination Space**

| Hyperparameter | | Range/Options |
| --- | --- | --- |
| Learning Rate (lr) | | [1e-5, 1e-4, 1e-3, 1e-2] |
| L2 Regularization (l2_lambda) | | [1e-6, 1e-5, 1e-4, 1e-3, 1e-2] |
| Batch Size | | [64, 128, 192, 256] |
| Hidden Dimensions | | [128, 256, 384, 512, 640, 768, 896, 1024, 1152, 1280, 1408, 1536, 1664, 1792, 1920, 2048] |
| Dropout Rate | | [0.1, 0.3, 0.5] |
| Number of Heads | | [1, 2, 3, 4, 5, 6, 7, 8] |
| Number of Layers | | [1, 2, 3] |
| Activation Function | | ['GELU', 'ReLU', 'LeakyReLU', 'Sigmoid', 'ELU', 'Tanh'] |
| Alpha (for LeakyReLU) | | [1e-3, 1e-2, 1e-1] |
| GAT Alpha | | [1e-3, 1e-2, 1e-1] |
| Convolution Layer | | ['GCNConv', 'GATConv', 'SAGEConv', 'GraphConv'] |
| Pooling Layer | ['TopKPooling', 'SAGPooling'] | |
| Global Pooling Layer | ['global_add_pool','global_mean_pool', 'global_max_pool'] | |
