# Supplementary material for: Graph-CRISPR: a gene editing efficiency prediction model based on graph neural network with integrated sequence and secondary structure feature extraction
Source: Brief Bioinform. 2025 Aug 15;26(4):bbaf410. doi: 10.1093/bib/bbaf410 (PMC12354951; doi:10.1093/bib/bbaf410)
Supplement: Table_S2_Final_Selection_of_Hyperparameter_bbaf410 [file table_s2_final_selection_of_hyperparameter_bbaf410.docx]

**Table S2 Final Selection of Hyperparameter**

| Name | Value |
| --- | --- |
| lr_values | 0.0001 |
| heads | 1 |
| hidden_dim | 1792 |
| layers | 3 |
| dropout | 0.1 |
| l2_lambda | 1e-05 |
| lr | 0.0001 |
| batch_size | 64 |
| alpha | 0.001 |
| Alpha | 0.001 |
| conv_layer | GCNConv |
| pool_layer | TopKPooling |
| global_pool_layer | global_mean_pool |
| activation | LeakyReLU |
