# Supplementary material for: Graph-CRISPR: a gene editing efficiency prediction model based on graph neural network with integrated sequence and secondary structure feature extraction
Source: Brief Bioinform. 2025 Aug 15;26(4):bbaf410. doi: 10.1093/bib/bbaf410 (PMC12354951; doi:10.1093/bib/bbaf410)
Supplement: Table_S3_Test_Dataset_Split_bbaf410 [file table_s3_test_dataset_split_bbaf410.docx]

**Table S3 Test Dataset Split**

| Dataset | Train-Val Set | Test set |
| --- | --- | --- |
| HL60 | 1765 | 312 |
| HELA | 6886 | 1215 |
| HCT116 | 3603 | 636 |
| WT | 47232 | 8341 |
| HF | 47150 | 8453 |
| ESP | 49823 | 8793 |
