# Supplementary material for: Graph-CRISPR: a gene editing efficiency prediction model based on graph neural network with integrated sequence and secondary structure feature extraction
Source: Brief Bioinform. 2025 Aug 15;26(4):bbaf410. doi: 10.1093/bib/bbaf410 (PMC12354951; doi:10.1093/bib/bbaf410)
Supplement: Table_S4_Statistical_analysis_for_the_WT_and_HCT116_bbaf410 [file table_s4_statistical_analysis_for_the_wt_and_hct116_bbaf410.docx]

**Table S4 Statistical analysis for the WT and HCT116 (threshold = 0.9).**

|  | **Mean Similarity (%)** | **Median Similarity (%)** | **Max**  **Similarity (%)** | **Min**  **Similarity (%)** |
| --- | --- | --- | --- | --- |
| **WT** | 3.2 | 0 | 100 | 0 |
| **HCT116** | 11.9 | 12 | 19 | 6 |
