# Supplementary material for: Graph-CRISPR: a gene editing efficiency prediction model based on graph neural network with integrated sequence and secondary structure feature extraction
Source: Brief Bioinform. 2025 Aug 15;26(4):bbaf410. doi: 10.1093/bib/bbaf410 (PMC12354951; doi:10.1093/bib/bbaf410)
Supplement: Table_S5_Analysis_for_the_Importance_of_Position_bbaf410 [file table_s5_analysis_for_the_importance_of_position_bbaf410.docx]

**Table S5 Analysis for the Importance of Position**

| No | target importance | source importance | impact score | Rank1 | Average Degree | Rank2 |
| --- | --- | --- | --- | --- | --- | --- |
| 1 | 0.8533 | 0.7068 | **0.7801** | **18** | **2.75** | **18** |
| 2 | 0.8422 | 0.7086 | **0.7754** | **1** | **2.70** | **2** |
| 3 | 0.7107 | 0.7082 | **0.7095** | **2** | **2.50** | **9** |
| 4 | 0.7114 | 0.707 | 0.7092 | 17 | 2.45 | 12 |
| 5 | 0.7099 | 0.7061 | 0.7080 | 15 | 2.35 | 14 |
| 6 | 0.7075 | 0.7067 | 0.7071 | 3 | 2.30 | 0 |
| 7 | 0.7087 | 0.7048 | 0.7068 | 14 | 2.30 | 5 |
| 8 | 0.7088 | 0.7043 | 0.7066 | 5 | 2.30 | 8 |
| 9 | 0.7064 | 0.7055 | 0.7060 | 4 | 2.30 | 10 |
| 10 | 0.7046 | 0.7053 | 0.7050 | 16 | 2.20 | 1 |
| 11 | 0.7065 | 0.7013 | 0.7039 | 13 | 2.20 | 17 |
| 12 | 0.7032 | 0.6993 | 0.7013 | 6 | 2.15 | 3 |
| 13 | 0.697 | 0.6941 | 0.6956 | 12 | 2.15 | 4 |
| 14 | 0.6928 | 0.6914 | 0.6921 | 7 | 2.15 | 7 |
| 15 | 0.683 | 0.6855 | 0.6843 | 11 | 2.15 | 13 |
| 16 | 0.6787 | 0.683 | 0.6809 | 8 | 2.05 | 6 |
| 17 | 0.6776 | 0.6806 | 0.6791 | 10 | 2.00 | 15 |
| 18 | 0.6772 | 0.6799 | 0.6786 | 9 | 1.95 | 19 |
| 19 | 0.4328 | 0.5808 | 0.5068 | 0 | 1.90 | 11 |
| 20 | 0.4143 | 0.5676 | 0.4910 | 19 | 1.85 | 16 |
|  | **Target-Source importance correlation**  **0.9198** | | | |  |  |
